# Effects of low glycemic index/load diets on metabolic and inflammatory markers in humans: a meta-analysis

**DOI:** 10.3389/fnut.2026.1836139

**Published:** 2026-06-12

**Authors:** Zijing Wu, Yao Xu, Linjie Qiu, Jixin Li, Yan Ren, Meijie Li, Chacha Zou, Jin Zhang

**Affiliations:** 1 Graduate School, Beijing University of Chinese Medicine, Beijing, China; 2 Xiyuan Hospital, China Academy of Chinese Medical Sciences, Beijing, China

**Keywords:** body weight loss, inflammatory factors, lipid metabolism, low glycemic index diets, low glycemic load diets, meta-analysis

## Abstract

**Objective:**

To evaluate the potential associations between low glycemic index (LGI) and low glycemic load (LGL) diets and variations in body weight, lipid profiles, and inflammatory markers through a meta-analysis.

**Methods:**

Systematic searches were conducted in PubMed, Cochrane Library, EMBASE, Web of Science, and Google Scholar for randomized controlled trials (RCTs) published through November 2025 (PROSPERO: CRD420251247827). Standardized mean differences (SMDs) with 95% confidence intervals (CIs) were estimated using random-effects or fixed-effects models.

**Results:**

21 RCTs (*n* = 1,265) were included. Meta-analytic evidence suggested that LGI/LGL diets were potentially associated with reductions in body weight (SMD = −1.09, *I^2^* = 92%), body mass index (BMI) (SMD = −1.39, *I^2^* = 93%), total cholesterol (TC) (SMD = −0.91, *I^2^* = 94%), triglycerides (TG) (SMD = −0.66, *I^2^* = 92%), and low-density lipoprotein cholesterol (LDL-C) (SMD = −1.40, *I^2^* = 95%), alongside an elevation in high-density lipoprotein cholesterol (HDL-C) (SMD = 0.67, *I^2^* = 88%). Significant attenuations were also identified in C-reactive protein (SMD = −0.86, *I^2^* = 91%), tumor necrosis factor-alpha (TNF-α) (SMD = −0.41, *I^2^* = 0%), interleukin-6 (IL-6) (SMD = −0.55, *I^2^* = 82%), and leptin (LEP) (SMD = −1.11, *I^2^* = 90%). However, no significant change was found for adiponectin (APN). Crucially, profound statistical heterogeneity was observed across the majority of metabolic outcomes.

**Conclusion:**

Current evidence suggests that LGI/LGL diets may favorably modulate body weight, lipid metabolism, and specific inflammatory markers. Nevertheless, these pooled estimates must be interpreted with extreme caution due to profound inter-study heterogeneity, suboptimal methodological quality of the included trials, and the statistical instability of TNF-α and IL-6. Consequently, this meta-analysis underscores the limitations of the existing literature rather than establishing definitive clinical efficacy.

## 1 Introduction

Dietary structures and patterns are recognized as key external factors influencing metabolic homeostasis (1). The “quality-quantity” characteristics of carbohydrates, as measured by the glycemic index (GI) and glycemic load (GL), critically modulate postprandial glucose and insulin responses, thereby shaping long-term energy balance and metabolic phenotypes (2). Previous studies suggest that diets targeting these parameters may offer a controllable approach to managing lipid metabolism and chronic obesity-associated risks (3).

Adipose tissue dysfunction, characterized by adipocyte hypertrophy and a sustained low-grade inflammatory state, is a hallmark of metabolic disease progression. This process involves the activation of pro-inflammatory macrophages and the release of cytokines such as tumor necrosis factor-alpha (TNF-α) and interleukin-6 (IL-6) (4). Although some research indicates that LGI/LGL diets may ameliorate these markers, evidence from randomized controlled trials (RCTs) remains inconsistent (5, 6). Consequently, a critical gap persists regarding the concurrent stability and robustness of the associations between LGI/LGL diets and diverse inflammatory markers and lipid subfractions, particularly given the high inter-study variability observed in previous trials.

To address this, the current meta-analysis systematically evaluates the impact of LGI/LGL diets on weight loss [body weight, body mass index (BMI)], lipid metabolism [total cholesterol (TC), triglycerides (TG), low-density lipoprotein cholesterol (LDL-C), high-density lipoprotein cholesterol (HDL-C)], and a broad range of inflammatory biomarkers [C-reactive protein (CRP), TNF-α, IL-6, adiponectin (APN), and leptin (LEP)]. By identifying potential sources of heterogeneity across different metabolic populations, we aim to provide comprehensive evidence to inform clinical nutritional strategies and to objectively clarify the potential value of these diets in managing chronic inflammation.

## 2 Methods

This meta-analysis was conducted in accordance with the Preferred Reporting Items for Systematic Reviews and Meta-Analyses (PRISMA 2020) statement (7). The study protocol has been prospectively registered with PROSPERO (Registration No.: CRD420251247827).

### 2.1 Literature search strategy

A comprehensive and systematic literature search was performed across five electronic databases: PubMed, Cochrane Library, EMBASE, Web of Science, and Google Scholar, from their inception through November 2025. No language restrictions were applied to ensure a comprehensive capture of available evidence. The search strategy employed a combination of Medical Subject Headings (MeSH) and free-text keywords related to the intervention and outcomes. The specific search queries encompassed four primary domains: (i) intervention parameters: “glycemic index,” “glycemic load,” “low-GI,” and “low-GL”; (ii) inflammatory markers: “inflammation,” “C-reactive protein,” “TNF-α,” and “interleukin-6”; (iii) metabolic indicators: “lipids,” “cholesterol,” “triglycerides,” “weight loss,” and “body mass index”; and (iv) study design: “randomized controlled trial” and “RCT.” Boolean operators (OR/AND) were used to combine these terms effectively within each database. The full, database-specific search strategies are documented in Supplementary Table 1. Additionally, manual screening of reference lists from included studies and pertinent review articles was conducted to identify further eligible publications.

### 2.2 Study selection

Studies were included in this analysis based on the following criteria: (1) Study Design: Only RCTs using either parallel or crossover designs were included; (2) Participants: Human adults aged 18 years or older. This included healthy individuals as well as those with specific metabolic conditions. In this meta-analysis, “metabolic conditions” were explicitly defined and categorized as overweight or obesity (BMI ≥ 25 kg/m^2^), type 2 diabetes mellitus (T2DM), metabolic syndrome (MS), and related states of insulin resistance or dyslipidemia. Patients with type 1 diabetes mellitus (T1DM) or unmanaged hypothyroidism were not specifically isolated as distinct subgroups due to limited original data. (3) Intervention: The experimental group received an LGI or LGL diet. (4) Control: The control group received high glycemic index/load (HGI/HGL) diets, standard healthy diets, or other habitual dietary patterns. (5) Outcomes: To ensure a comprehensive assessment of cardio-metabolic health, studies were required to report a multi-dimensional profile incorporating concurrent data on anthropometric markers (e.g., body weight, BMI), lipid profiles (e.g., TC, TG, LDL-C), and systemic inflammatory biomarkers (e.g., CRP, IL-6).

The exclusion criteria were as follows: (1) Non-RCT designs, including observational studies, case reports, reviews, and animal or *in vitro* experiments; (2) Specific populations such as pregnant or lactating women; (3) Patients with severe acute or chronic conditions that independently impact systemic inflammation, such as active infections, malignant tumors, or end-stage organ failure; (4) Studies where dietary interventions were combined with pharmacological weight-loss medications or bariatric surgery; (5) Studies with insufficient data for calculating effect sizes (e.g., missing Mean and SD) where data remained unavailable after contacting authors.

### 2.3 Data extraction and quality assessment

Following the removal of duplicates, two independent reviewers (ZW and YX) screened the remaining records based on titles and abstracts. Subsequently, the reviewers selected potentially eligible full-text articles for final inclusion. Any discrepancies between the two reviewers regarding study eligibility or data extraction were resolved through consensus via discussion or, if necessary, by consulting a third senior reviewer (JZ). The two reviewers extracted data including the title, first author, publication year, study design, participant characteristics (health status, sex, age, BMI), sample sizes for the LGI/LGL and control groups, specific GI/GL values for each group, study duration, and outcome measures. For crossover trials, given the absence of consistently reported intra-individual correlation coefficients across the primary literature, data from the terminal phase of each intervention period were extracted and conservatively evaluated as independent parallel groups. Furthermore, the adequacy of washout periods in the primary trials was systematically verified to minimize potential carry-over effects.

Adhering to the recommendations in the latest version of the Cochrane Handbook for Systematic Reviews of Interventions (Version 6.5, updated October 2024) (8), two authors independently assessed the risk of bias in the included RCTs. Evaluations encompassed seven domains: random sequence generation, allocation concealment, blinding of participants and personnel, blinding of outcome assessment, incomplete outcome data, selective reporting, and other potential biases. Each item was categorized as having a low, high, or unclear risk of bias. Additionally, STATA (version 15.1) was employed to generate funnel plots to identify the presence of publication bias.

### 2.4 Statistical analysis

Statistical synthesis was performed using Review Manager (RevMan, version 5.4) to generate forest and funnel plots. To ensure methodological uniformity across a multidimensional profile of continuous outcomes—and specifically to account for the substantial baseline variance in anthropometric characteristics (ranging from normal weight to morbid obesity), effect sizes for all parameters, including body weight and BMI, were uniformly expressed as standardized mean differences (SMDs) with 95% confidence intervals (CIs). Statistical heterogeneity was evaluated using the Cochran’s *Q* test and *I*^2^ statistic. A random-effects model was selected if *I*^2^ ≥ 50% or *p <* 0.05, indicating significant heterogeneity; otherwise, a fixed-effects model was applied. This predefined approach was strictly followed to ensure objective model selection based on statistical indicators of heterogeneity (*p*-value and *I*^2^), although results from fixed-effects models in analyses with limited study numbers were cross-verified with sensitivity analyses to ensure robustness. Despite the clinical diversity of the included populations (healthy, overweight/obese, and T2DM), pooling was performed to evaluate the broad metabolic impact of LGI/LGL diets, with potential population-specific effects further explored through predefined subgroup analyses. To explore the sources of heterogeneity, predefined subgroup analyses were conducted based on: (1) Metabolic health status (overweight/obesity, T2DM/MS, or healthy); (2) Control dietary patterns (HGI/HGL diets vs. other conventional diets); and (3) Intervention duration (short-term <12 weeks vs. long-term ≥12 weeks). Sensitivity analysis was performed using the leave-one-out method to assess the robustness of the results. Funnel plots were used to evaluate publication bias. In cases where standard deviations were not reported in the original literature, they were estimated using the calculator provided in the Cochrane Handbook. A *p*-value <0.05 was considered statistically significant.

## 3 Results

### 3.1 Study selection

**Figure 1:**
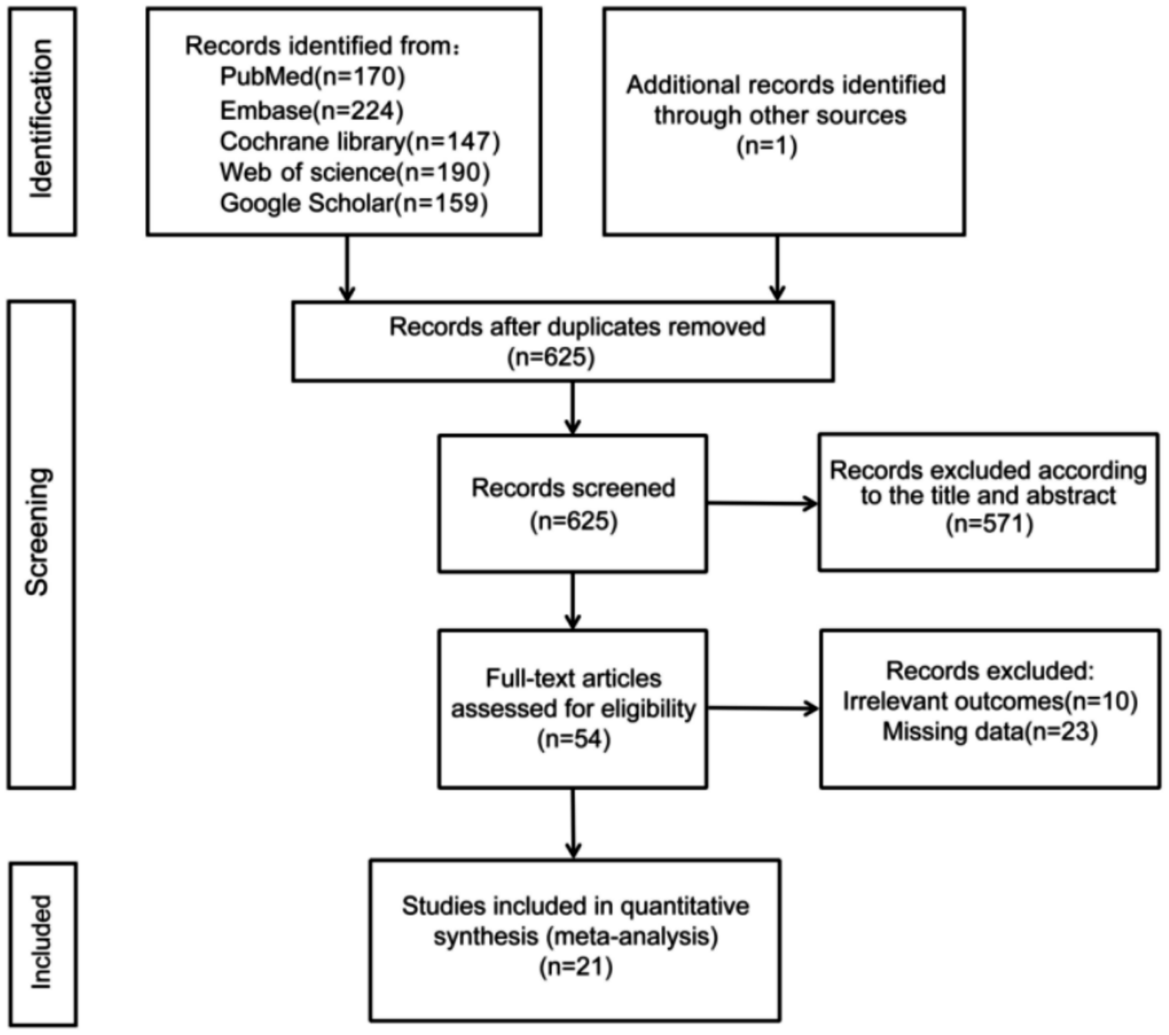
Flow diagram of the literature screening process.

The systematic literature screening process is detailed in Figure 1. A total of 890 records were initially identified through database searches, alongside 1 additional record identified through manual searching. After removing 266 duplicate records, 625 distinct records underwent preliminary screening of titles and abstracts. Based on the pre-defined inclusion and exclusion criteria, 571 records were excluded at this stage. After a rigorous full-text evaluation of the 54 remaining candidate studies, 33 were excluded for reasons such as irrelevant outcomes (*n* = 10) or missing data (*n* = 23). Ultimately, 21 RCTs encompassing 1,265 participants were deemed eligible for inclusion in the meta-analysis.

### 3.2 Study characteristics

The baseline characteristics of the included study populations are summarized in Table 1. All 21 included RCTs were published between 2002 and 2024, with intervention durations spanning from 4 weeks to 1 year. Regarding the study architecture, 17 trials (9–25) utilized a parallel-group design, whereas the remaining 4 trials (26–29) employed a crossover design. In terms of the participants’ metabolic conditions, 11 studies (10, 12–16, 19, 22, 24, 28, 29) focused on overweight or obese individuals, 6 studies (9, 11, 17, 20, 25, 27) involved patients with comorbid MS or T2DM, 3 studies (18, 21, 26) recruited healthy cohorts, and 1 study (23) specifically investigated populations with MS. It is worth noting that within these primary classifications, some individual trials included participants with concurrent metabolic conditions (e.g., underlying insulin resistance in the obese cohort). When conducting the subgroup analyses, participants were broadly stratified into ‘metabolic conditions’ versus ‘healthy individuals’. Within the ‘metabolic conditions’ subgroup, the specific comorbidities were predominantly composed of T2DM, obesity-associated insulin resistance, and MS, as detailed in Table 1.

**Table 1:** Basic characteristics of the included studies.

First author, year	Country/Region	Study design	Baseline characteristics	Intervention			Sample size (*n*)	Duration	Outcomes
				Experimental group	Control group	Other intervention group			
Argiana, 2015 (9)	Greece	Parallel	Males and postmenopausal females with T2DM, aged 40–65 years; BMI: 25–40	LGI (GI: 19–49)	Medium GI (GI: 60–65)	NA	30/31	12 weeks	Weight (short for body weight), BMI, TC, TG, HDL-C, LDL-C, hs-CRP, IL-6, APN, LEP
Becker, 2015 (10)	Brazil	Parallel	Overweight or obese infertile women, aged 18–35 years; BMI: 25–40	LGI (GI < 55), LGL (GL < 80)	usual diets (unrestricted)	NA	14/12	5 weeks	Weight, TC, TG, LDL-C, HDL-C, BMI, LEP
Bouché, 2002 (26)	France	Crossover	Healthy men, aged 46 ± 3 years	LGI diet (GI = 41.0 ± 1.0%)	HGI diet (GI = 71.3 ± 1.3%)	NA	11	5 weeks	Weight, TC, TG, LEP
Myette-Côté, 2018 (27)	Britain	Crossover	Males and females with T2DM, aged 48–72 years	Low-fat LGI diet (GI = 40)	Low-carb high-fat diet (LC)	LC diet + exercise (15-min walks)	16	6 weeks	Weight, TG, TNF-α, IL-6
Gomes, 2016 (11)	Brazil	Parallel	Males and premenopausal females with T2DM, aged 42.4 ± 5.1 years; BMI: 29.2 ± 4.8	LGI diet (GI: 35.8 ± 3.3)	HGI diet (GI: 74.1 ± 2.9)	NA	10/10	30 days	BMI, TC, TG, HDL-C, APN, CRP
Mehrabani, 2012 (12)	Iran	Parallel	Overweight or obese women, aged 20–40 years; BMI: 25–38	Modified hypocaloric diet (prohibition of foods with GL > 20)	Conventional hypocaloric diet (CHCD)	NA	26/23	12 weeks	Weight, TG, TC, HDL-C, LDL-C, TNF-α, IL-6, hs-CRP, APN
Abete, 2008 (13)	Spain	Parallel	Obese males and females, aged 36 ± 7 years; BMI: 32.5 ± 4.3	LGI diet (GI: 40–45)	HGI diet (GI: 60–65)	NA	16/16	8 weeks	Weight, BMI, LEP, TC, TG, HDL-C, LDL-C
McMillan-Price, 2006 (14)	Australia	Parallel	Overweight males and females, aged 18–40 years; BMI ≥ 25	LGI diet (GI: 75)	HGI diet (GI: 127)	NA	32/32	12 weeks	Weight, TC, TG, HDL-C, LDL-C, LEP, CRP
Juanola-Falgarona, 2014 (15)	Spain	Parallel	Overweight or obese men and women, aged 30–60 years; MI: 27–35	LGI diet (GI: 34)	HGI diet (GI: 62)	Low-fat diet (LF)	41/40/40	6 months	Weight, TC, TG, HDL-C, LDL-C, CRP, IL-6, APN, LEP
Krog-Mikkelsen, 2011 (16)	Denmark	Parallel	Overweight females, aged 20–40 years; BMI: 25–30	LGI diet (GI: 79)	HGI diet (GI: 103)	NA	14/15	10 weeks	Weight, BMI, LEP
Bozzetto, 2021 (17)	Italy	Parallel	Males and females with T2DM	LGI diet (GI: 48) + Exercise (Ex)	Monounsaturated fatty acids (MUFA) diet (GI: 60) + Exercise (Ex)	NA	7/9	8 weeks	BMI, LEP, TC, TG, HDL-C, LDL-C
Marsh, 2010 (18)	Australia	Parallel	Healthy women, aged 18–60 years; BMI ≤ 25	LGI diet (GI: 40, GL: 74)	Conventional healthy diet (CHD) (GI: 74, GL: 109)	NA	50/46	4 weeks	Weight, BMI, TC, TG, HDL-C, LDL-C, CRP
Melanson, 2012 (19)	America	Parallel	Overweight or obese men and women, aged 38.7 ± 6.7 years; BMI: 31.8 ± 2.2	LGI diet (GI: 42.43 ± 7.35, GL: 44.75 ± 27.86)	Portion-controlled (PC) plan	Low energy density (LED) diet (GI: 40.15 ± 8.64, GL: 54.39 ± 30.14)	59/57/41	12 weeks	Weight, BMI, TG, HDL-C, CRP
Nivedita, 2020 (20)	India	Parallel	T2DM men and women, aged 35–65 years	LGI diet	HGI diet	NA	40/40	24 weeks	Weight, BMI, TG, TC, HDL-C, LDL-C, hs-CRP
Gögebakan, 2011 (21)	Europe	Parallel	Healthy men and women, aged <65 years	LGI diet (GI: 45)	HGI diet (GI: 60)	NA	35/48	26 weeks	Weight, TG, TC, HDL-C, LDL-C, hs-CRP
Pereira, 2004 (22)	America	Parallel	Overweight or obese men and women, aged 18–40 years; BMI > 27	LGI diet	Low-fat diet	NA	22/17	1 year	Weight, TG, LDL-C, HDL-C, CRP
Rizkalla, 2012 (28)	France	Crossover	Obese men and women, aged 45.0 ± 2.4 years; BMI: 31.86 ± 1.30	Energy-restricted LGI diet compensated by protein (LC-P-LGI)	Conventional diet (LC-CONV)	NA	13	16 weeks	Weight, TC, TG, LDL-C, HDL-C, hs-CRP, TNF-α, IL-6, APN, LEP
Giacco, 2013 (23)	Finland	Parallel	Men and women with metabolic syndrome, aged 40–65 years	Wholegrain LGI group (GI: 46)	Control HGI group (GI: 72)	NA	61/62	12 weeks	Weight, BMI, TC, TG, HDL-C, LDL-C, hs-CRP, TNF-α, IL-6
Shikany, 2009 (29)	America	Crossover	Overweight or obese men, aged 25.0 ± 2.8 years; BMI: 29.5 ± 4.3	LGI diet (GI: 49.5 ± 3.3, GL: 158.3 ± 12.8)	HGI diet (GI: 75.0 ± 4.2, GL: 245.5 ± 11.4)	NA	24	12 weeks	Weight, BMI, TC, LDL-C, HDL-C, TG, CRP, IL-6, TNF-α
Suder, 2024 (24)	Poland	Parallel	Abdominally obese males, aged 34.7 ± 5.5 years; WC: 110.3 ± 8.5 cm	Exercise + High protein LGI diet (EDG)	Exercise group (EG)	Control group (CG)	16/16/12	6 weeks	Weight, TC, LEP
Wolever, 2008 (25)	Canada	Parallel	T2DM men and women, aged 35–75 years; BMI: 24–40	LGI diet (GI: 55)	HGI diet (GI: 63)	Low cholesterol diet (CHO)	56/51/54	1 year	Weight, TC, TG, HDL-C, LDL-C, CRP

NA, not applicable; indicates the absence of a third intervention group in two-arm studies or the non-existence of a specific subgroup for a particular outcome. For parallel designs, sample sizes are presented as experimental/control (/ other intervention). For crossover designs, the number represents the total number of participants who completed the trial. Co-interventions (e.g., exercise or specific macronutrient adjustments) were consistently applied across groups within the respective trials to minimize potential confounding effects.

### 3.3 Risk of bias

As depicted in Table 2, all 21 included trials were categorized as having an unclear risk of selection bias concerning allocation concealment, primarily due to insufficient reporting. While all 21 RCTs provided comprehensive descriptions of their random sequence generation methods, documentation regarding allocation concealment remained deficient across all 21 studies (9–29). Critically, 20 RCTs were assessed as being at high risk for detection bias (blinding of outcome assessment) due to implementation deficiencies (9–24, 26–29). 9 RCTs were identified as having a high risk of performance bias related to the blinding of participants and personnel (10, 13, 18–20, 22–24, 27), and a high risk of attrition bias due to incomplete outcome data was noted in one study (18).

**Table 2:** Risk of bias assessment.

First author, year	Random sequence generation	Allocation concealment	Blinding of participants and personnel	Blinding of outcome assessment	Incomplete outcome data	Selective reporting	Other bias
Argiana, 2015 (9)	Low	Unclear	Low	High	Low	Low	Low
Becker, 2015 (10)	Low	Unclear	High	High	Low	Low	Low
Bouché, 2002 (26)	Low	Unclear	Low	High	Low	Low	Low
Myette-Côté, 2018 (27)	Low	Unclear	High	High	Low	Low	Low
Gomes, 2016 (11)	Low	Unclear	Low	High	Low	Low	Low
Mehrabani, 2012 (12)	Low	Unclear	Low	High	Low	Low	Low
Abete, 2008 (13)	Low	Unclear	High	High	Low	Low	Low
McMillan-Price, 2006 (14)	Low	Unclear	Low	High	Low	Low	Low
Juanola-Falgarona, 2014 (15)	Low	Unclear	Low	High	Low	Low	Low
Krog-Mikkelsen, 2011 (16)	Low	Unclear	Low	High	Low	Low	Low
Bozzetto, 2021 (17)	Low	Unclear	Low	High	Low	Low	Low
Marsh, 2010 (18)	Low	Unclear	High	High	High	Low	Low
Melanson, 2012 (19)	Low	Unclear	High	High	Low	Low	Low
Nivedita, 2020 (20)	Low	Unclear	High	High	Low	Low	Low
Gögebakan, 2011 (21)	Low	Unclear	Low	High	Low	Low	Low
Pereira, 2004 (22)	Low	Unclear	High	High	Low	Low	Low
Rizkalla, 2012 (28)	Low	Unclear	Low	High	Low	Low	Low
Giacco, 2013 (23)	Low	Unclear	High	High	Low	Low	Low
Shikany, 2009 (29)	Low	Unclear	Low	High	Low	Low	Low
Suder, 2024 (24)	Low	Unclear	High	High	Low	Low	Low
Wolever, 2008 (25)	Low	Unclear	Low	Low	Low	Low	Low

### 3.4 Meta-analysis results

#### 3.4.1 Weight loss indicators (body weight and BMI)

**Figure 2:**
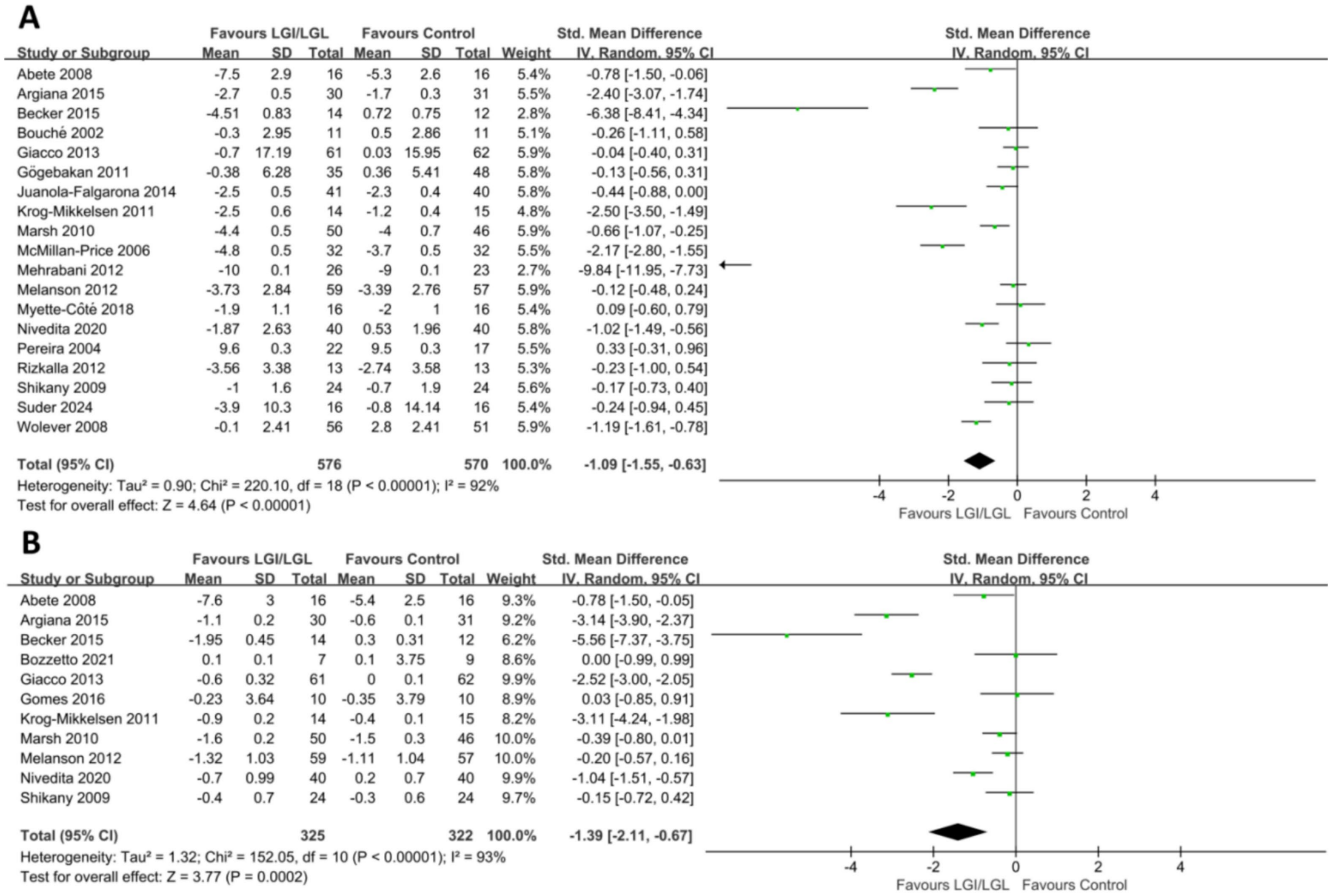
Forest plots for the effect of LGI/LGL diets on weight loss indicators: **(A)** body weight; **(B)** BMI.

Substantial statistical heterogeneity was observed for both body weight and BMI outcomes (*I^2^* = 92 and 93%, respectively; both *p <* 0.001), necessitating the use of a random-effects model. Meta-analysis results suggested that LGI/LGL diets were associated with significant reductions in body weight across 19 trials (9, 10, 12–16, 18–29) (SMD = −1.09; 95% CI: −1.55, −0.63; *p* < 0.001) and BMI across 11 trials (9–11, 13, 16–20, 23, 29) (SMD = −1.39; 95% CI: −2.11, −0.67; *p* < 0.001). The corresponding forest plots are presented in Figures 2A,B.

#### 3.4.2 Lipid metabolism indicators (TG, TC, LDL-C, and HDL-C)

**Figure 3:**
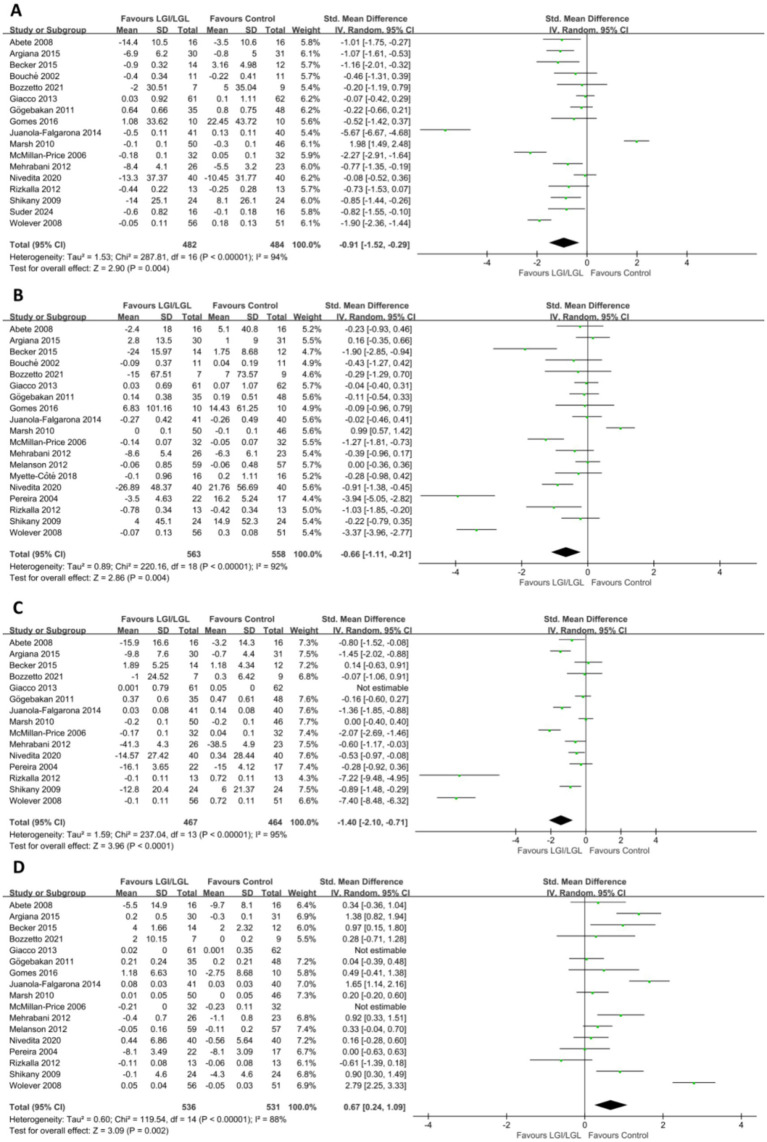
Forest plots for the effect of LGI/LGL diets on lipid metabolism indicators: **(A)** TC; **(B)** TG; **(C)** LDL-C; **(D)** HDL-C.

Regarding lipid profiles, significant statistical heterogeneity was found across all outcomes (*I^2^* range: 88–95%, all *p* < 0.001); consequently, random-effects models were employed for the pooled analysis. Results indicated potential associations between LGI/LGL diets and lower levels of TG [19 trials (9–15, 17–23, 25–29); SMD = −0.66; 95% CI: −1.11, −0.21; *p* = 0.004; *I^2^* = 92%], TC [17 trials (9–15, 17, 18, 20, 21, 23–26, 28, 29); SMD = −0.91; 95% CI: −1.52, −0.29; *p* = 0.004; *I^2^* = 94%], and LDL-C [15 trials (9, 10, 12–15, 17, 18, 20–23, 25, 28, 29); SMD = −1.40; 95% CI: −2.10, −0.71; *p* < 0.001; *I^2^* = 95%]. Simultaneously, an elevation in HDL-C was observed [17 trials (9–15, 17–23, 25, 28, 29); SMD = 0.67; 95% CI: 0.24, 1.09; *p* = 0.004; *I^2^* = 88%], as illustrated in Figures 3A–D.

#### 3.4.3 Inflammatory biomarkers (CRP, TNF-α, IL-6, APN, and LEP)

**Figure 4:**
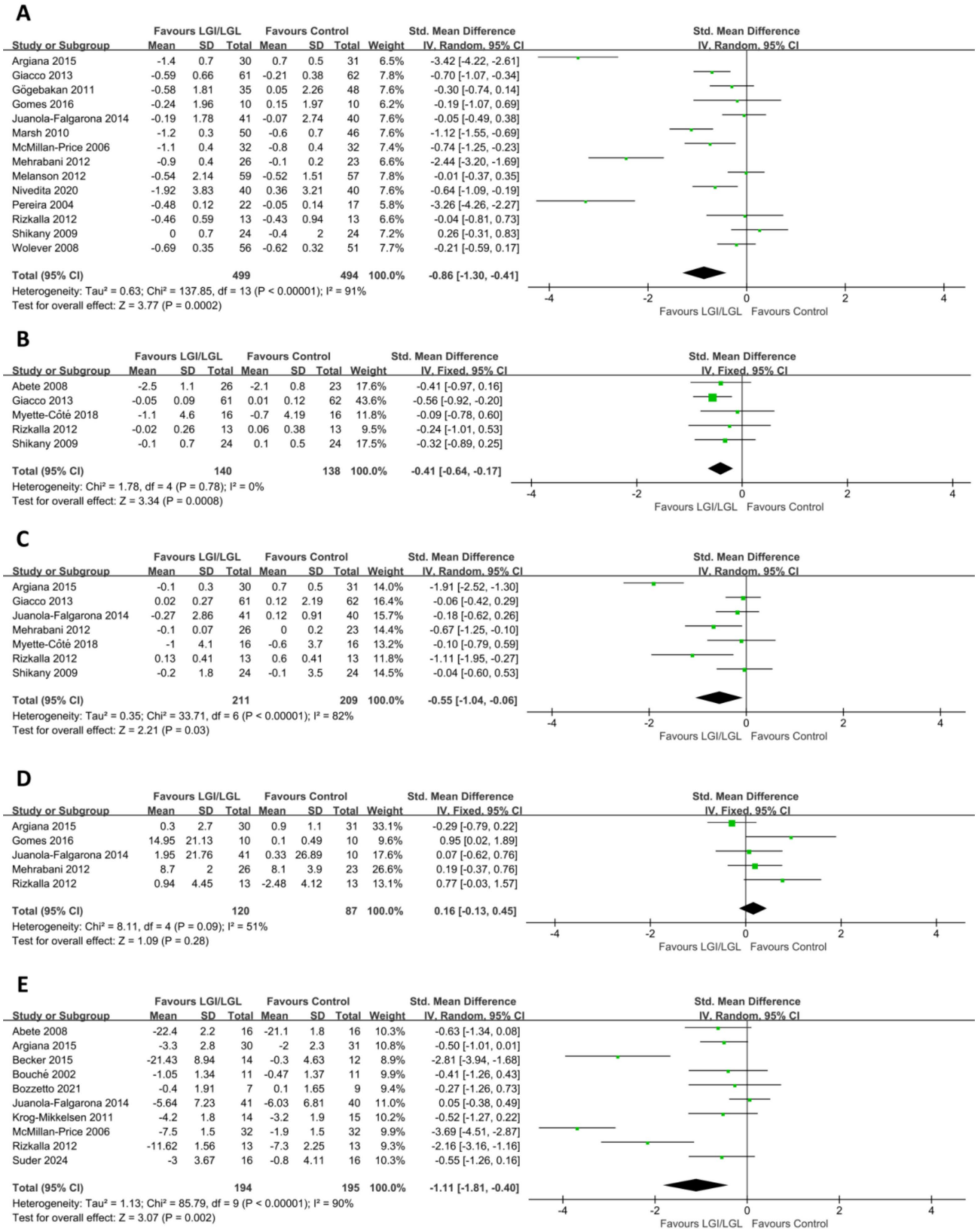
Forest plots for the effect of LGI/LGL diets on lipid metabolism indicators: **(A)** CRP; **(B)** TNF-α; **(C)** IL-6; **(D)** APN; **(E)** LEP.

For inflammatory biomarkers, random-effects models were applied for CRP, IL-6, and LEP due to high heterogeneity (*I^2^* = 91, 82, and 90%, respectively), while fixed-effects models were utilized for TNF-α (*I^2^* = 0%) and APN (*I^2^* = 51%). The meta-analysis revealed that LGI/LGL diets were associated with significant reductions in CRP [14 trials (9, 11, 12, 14, 15, 18–23, 25, 28, 29); SMD = −0.86; 95% CI: −1.30, −0.41; *p* < 0.001], TNF-α [5 trials (12, 23, 27–29); SMD = −0.41; 95% CI: −0.64, −0.17; *p* < 0.001], IL-6 [7 trials (9, 12, 15, 23, 27–29); SMD = −0.55; 95% CI: −1.04, −0.06; *p* = 0.03], and LEP [10 trials (9, 10, 13–17, 24, 26, 28); SMD = −1.11; 95% CI: −1.81, −0.40; *p* = 0.002]. No significant difference was found for APN levels [5 trials (9, 11, 12, 15, 28); SMD = 0.16; 95% CI: −0.13, 0.45; *p* = 0.28], as illustrated in Figures 4A–E. Notably, sensitivity analyses indicated that the pooled results for TNF-α and IL-6 were highly sensitive to the exclusion of individual studies, suggesting pronounced statistical instability in these specific inflammatory outcomes.

### 3.5 Subgroup analysis

To further explore the potential sources of clinical and statistical heterogeneity, subgroup analyses were performed, stratified by the health status of the study populations and the dietary patterns of the control groups. For the LDL-C outcome, the subgroup analysis delineated significant between-group differences concerning participant health status (*p* = 0.002). In contrast, no statistically significant between-group heterogeneity was identified across the remaining subgroups (*p* > 0.05). These findings imply that baseline health status may be the primary driver of the observed heterogeneity in LDL-C outcomes. Detailed results of these analyses are summarized in Table 3. The results of subgroup analysis for other groups are presented in the Supplementary Tables 4–14.

**Table 3:** Subgroup analysis for LDL-C.

Subgroup	Number of studies	SMD (95% CI)	*p*-value	*I^2^* (%)	Model	Between-group *p*-value
Health status of the study population						0.002
Metabolic conditions	5	−1.85 [−3.51,-0.19]	0.03	98	Random-effects	
Overweight/obese	8	−1.22 [−1.91,-0.52]	<0.001	88	Random-effects	
Healthy individuals	2	−0.07 [−0.37,0.22]	0.62	0	Fixed-effects	
Dietary patterns of the control group						0.12
HGI/HGL diets	9	−1.76 [−2.74,-0.78]	<0.001	96	Random-effects	
Other dietary types/other types of diets	6	−0.75 [−1.58,0.09]	0.08	88	Random-effects	

SMD, standardized mean difference; CI, confidence interval; NA, not applicable. “NA” indicates the non-existence of a specific subgroup for this outcome, or that heterogeneity and between-group *p*-values could not be calculated due to an insufficient number of studies (*n* < 2). *p* < 0.05 was considered statistically significant.

### 3.6 Sensitivity analysis

Sensitivity analysis was conducted using the leave-one-out method to assess the stability of the pooled estimates. The results indicated that the findings for the TNF-α and IL-6 groups exhibited suboptimal stability. Specifically, the effect size for TNF-α became statistically non-significant after the exclusion of the study by Giacco et al. (23) [SMD = −0.29; 95% CI: −0.60, 0.03; *p* = 0.08]. Similarly, the findings for IL-6 lost statistical significance upon the exclusion of the study by Mehrabani et al. (12) [SMD = −0.54; 95% CI: −1.11, 0.03; *p* = 0.06]. Furthermore, statistical heterogeneity decreased markedly following the exclusion of the study by Argiana et al. (9), suggesting that this trial was a primary contributor to the observed heterogeneity. In the APN group, excluding the study by Argiana et al. (9) shifted the pooled result from non-significant to significant [SMD = 0.38; 95% CI: 0.03, 0.74; *p* = 0.03] and substantially attenuated heterogeneity. Collectively, these findings imply that the study by Argiana et al. (9) exerted a notable impact on the robustness and heterogeneity of both the IL-6 and APN outcomes. Because the statistical significance of TNF-α and IL-6 was heavily dependent on individual trials, these specific inflammatory outcomes cannot be considered robust and must be interpreted with extreme caution.

### 3.7 Publication bias

**Figure 5:**
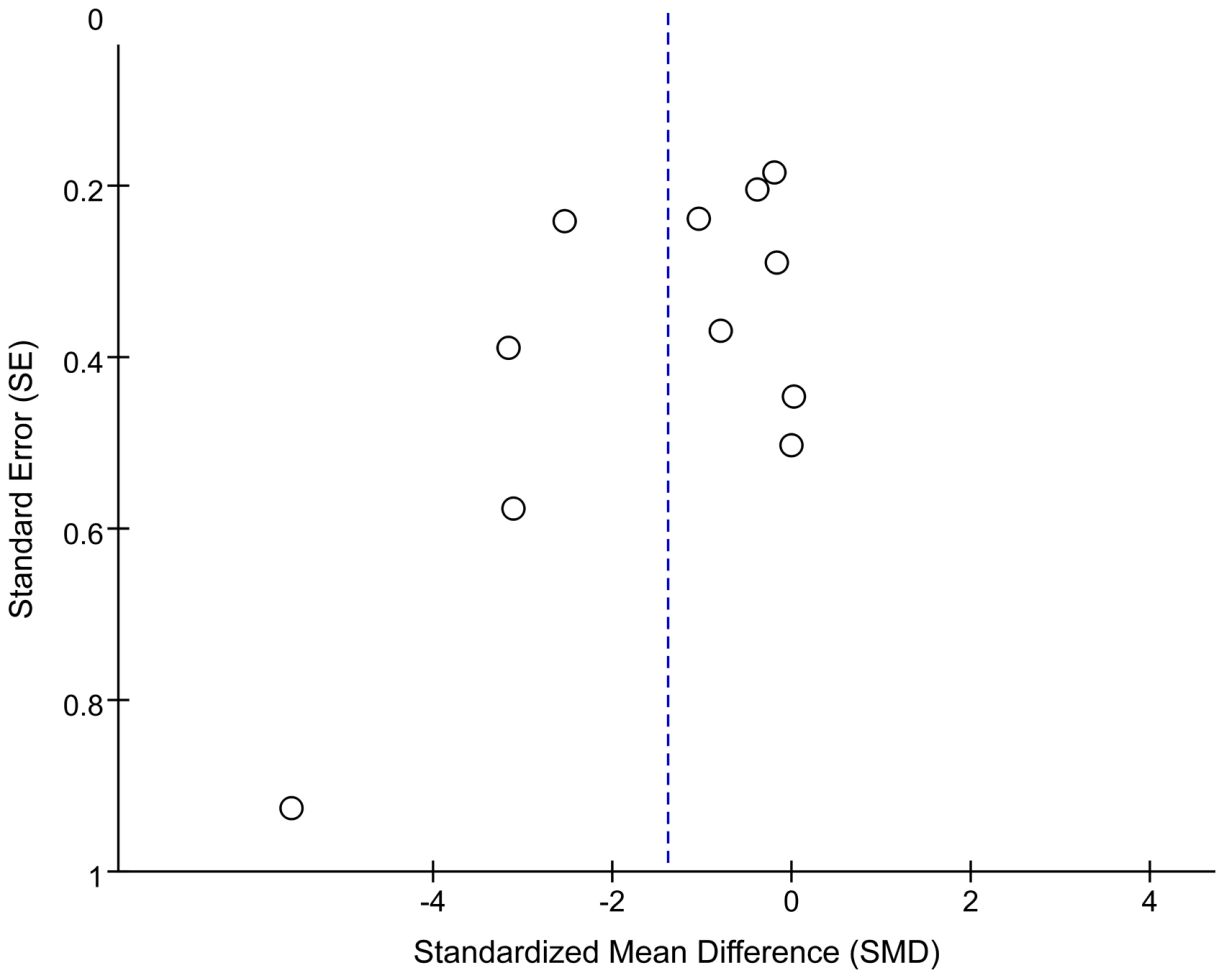
Publication bias of BMI.

Following Cochrane guidelines (recommending assessment only for outcomes with ≥10 trials), publication bias was evaluated for BMI, body weight, lipid profiles, CRP, and LEP. For BMI, the visually symmetrical funnel plot (Figure 5) was corroborated by Begg’s (*p* = 0.559) and Egger’s tests (*p* = 0.095) via STATA 15.1, confirming no significant publication bias. While quantitative statistical testing was prioritized for this primary anthropometric indicator, funnel plots for the other eligible outcomes similarly displayed symmetrical distributions. These additional plots (Supplementary Figures 5–12) suggest that our overall findings are robust and unlikely to be significantly influenced by publication bias.

## 4 Discussion

This meta-analysis of 21 RCTs (*n* = 1,265) suggests that LGI/LGL diets are potentially associated with favorable modulations in body weight, BMI, lipid profiles (TG, TC, LDL-C, HDL-C), and specific systemic inflammatory biomarkers (CRP, TNF-α, IL-6, and LEP). APN levels remained unchanged. While these findings expand upon prior systematic reviews that reported neutral or conflicting results (6, 30), leave-one-out sensitivity analyses revealed marked instability in the TNF-α and IL-6 estimates, mandating a highly cautious interpretation. Our synthesis indicates potential favorable trends regarding cardio-metabolic parameters, aligning with recent meta-analyses targeting glycemic-controlled dietary interventions (31). However, interpreting these findings requires the strict avoidance of exclusive causality assumptions. Inflammatory markers, particularly CRP, exhibit high pleiotropy and are profoundly influenced by unmeasured confounders, such as emotional stressors, subclinical infections, and diverse autoimmune states (32). Consequently, the observed reductions likely reflect a complex, multifactorial biological phenomenon rather than a singular, diet-driven outcome.

Biochemically, restricting glycemic load alters the metabolic environment of adipose tissue. Blunting postprandial glycemic excursions likely downregulates 1-acylglycerol-3-phosphate-O-acyltransferase, limiting hepatic VLDL-C and TG production, while potentially suppressing HMG-CoA reductase to attenuate endogenous cholesterol synthesis (33, 34). Additionally, LGI/LGL diets ameliorate the pro-inflammatory microenvironment, characterized by M1-macrophage infiltration and impaired insulin signaling (35, 36), secondary to weight and visceral fat reduction. This loss of adiposity decreases leptin and restores adiponectin expression, synergistically exerting anti-inflammatory and insulin-sensitizing effects (37, 38). Nevertheless, lipid and inflammatory markers are modulated by genetic variability, medication, allostatic load, and stress. Therefore, these improvements represent potential associations, not a definitive cause-and-effect relationship.

It is imperative to emphasize that the profound statistical heterogeneity (*I^2^* > 90%) observed herein reflects the inherent, unavoidable clinical diversity characteristic of free-living dietary interventions. Rather than artificially manipulating inclusion criteria to lower statistical variance, a practice that risks introducing severe selection bias, we maintain that this heterogeneity accurately and objectively represents the current, imperfect state of evidence in nutritional epidemiology. Several critical factors drive this variance: First, pooling participants with divergent metabolic phenotypes (e.g., healthy cohorts versus those with severe insulin resistance) inherently drives effect size variance, as baseline glycemic control and anthropometry dictate an individual’s sensitivity to GI/GL modifications (39, 40). This variance is further compounded by complex gene-diet interactions, where genetic susceptibility significantly dictates individual weight loss and lipid metabolism trajectories (41). Unmeasured confounders, such as fiber intake, food matrices, and baseline inflammatory states, likely exacerbate this heterogeneity further. Second, variations in control diets and unstandardized co-interventions differentially impact markers like LDL-C (42). In routine clinical practice, LGI diets are frequently combined with higher fat intakes. While carbohydrate restriction benefits specific populations like T1DM (43), unmonitored saturated fat intake can paradoxically elevate LDL-C and exacerbate cardiovascular risk. Importantly, the clinical utility of LGI/LGL diets extends beyond the management of T2DM and obesity. Emerging evidence indicates that such carbohydrate-restricted dietary approaches offer substantial benefits for patients with T1DM as well. When implemented in conjunction with standard pharmacological therapies, low-carbohydrate and LGI interventions have been shown to significantly contribute to the reduction of glycated hemoglobin (HbA1c) levels and the lowering of daily insulin dose requirements (44). This highlights the broad metabolic applicability of glycemic-controlled diets across different pathophysiological states. Finally, methodological discrepancies in assay techniques contribute to measurement error, particularly for adiponectin (45, 46).

Consequently, our findings represent potential associations rather than definitive efficacy. As detailed in Table 2, the included populations encompass diverse geographical regions. This ethnic diversity strongly implies that genetic variability acts as an unmeasured confounder for lipid profiles. Furthermore, socioeconomic characteristics profoundly shape long-term lifestyle habits (e.g., physical activity, psychological stress), exerting substantial baseline effects on metabolic and inflammatory loads. Several methodological limitations must be acknowledged: (1) Inconsistent intervention definitions and highly varied control diets limit direct inter-study comparability. (2) Discrepancies in intervention durations (4 weeks to 1 year) constrain short- versus long-term applicability. Notably, while short-term interventions may quickly modulate postprandial lipids, significantly longer durations are typically required to detect stable, physiological reductions in adiposity and systemic inflammation. (3) High population heterogeneity and unassessed comorbidities: Including cohorts ranging from healthy individuals to those with advanced T2DM, limits generalizability. The inadequate description of underlying conditions (e.g., hypertension, chronic kidney disease, depression, or gastrointestinal disorders) in the original trials significantly obscures the physiological efficacy of LGI/LGL diets. (4) The overall methodological quality of the original literature is a major concern. Crucially, allocation concealment was unclear across all 21 included trials, and the vast majority of studies suffered from a high risk of detection bias. These structural flaws significantly compromise the reliability of the pooled estimates. (5) Limited sample sizes in certain subgroups and the marked statistical sensitivity of specific markers (TNF-α, IL-6) severely diminish the robustness of these inflammatory endpoints. Because their statistical significance was heavily dependent on individual trials, these specific outcomes cannot be considered robust. (6) Varied and often insufficient control of residual confounders, particularly precise energy intake and physical activity. (7) Limitations in advanced statistical analysis: Inconsistent reporting of continuous covariates precluded the execution of meta-regression, limiting our ability to quantitatively partition the exact sources of heterogeneity. Furthermore, pooling effect sizes utilizing SMDs abstracts absolute biological values, potentially hindering the direct clinical application of these results regarding exact, targetable lipid thresholds.

Future research should: (1) Utilize standardized GI/GL protocols reporting key co-variables (e.g., fiber, energy intake). (2) Design long-term, adequately powered RCTs controlling for physical activity and medication. (3) Conduct stratified studies across diverse ethnic and metabolic phenotypes to verify inflammatory marker consistency.

## 5 Conclusion

In conclusion, while pooled estimates suggest that LGI/LGL diets may be associated with modest improvements in body weight, lipid profiles, and certain systemic inflammatory markers, the current body of evidence lacks the methodological robustness required to establish definitive clinical efficacy. The reliability and clinical interpretability of these findings are substantially constrained by profound statistical heterogeneity (often >90%), widespread methodological flaws within the original trials (particularly unclear allocation concealment and high detection bias), and the marked statistical instability of TNF-α and IL-6 estimates. Therefore, the present meta-analysis objectively highlights the imperfect state of the available literature rather than confirming an absolute dietary solution. Future dietary guidelines should cautiously weigh these limitations, and subsequent research must prioritize highly rigorous, well-powered RCTs with transparent methodological reporting to confirm any true, causal metabolic benefits.

## Data Availability

The original contributions presented in the study are included in the article/Supplementary material, further inquiries can be directed to the corresponding author.
