# Supplementary material for: Effects of low glycemic index/load diets on metabolic and inflammatory markers in humans: a meta-analysis
Source: Front Nutr. 2026 Jun 12;13:1836139. doi: 10.3389/fnut.2026.1836139 (PMC13303483; doi:10.3389/fnut.2026.1836139)
Supplement: Supplementary file 1 [file Data_Sheet_1.DOCX]

Supplementary Material

# Supplementary Data

Supplementary Material should be uploaded separately on submission. Please include any supplementary data, figures, and/or tables.

Supplementary material is not typeset, so please ensure that all information is clearly presented, the appropriate caption is included in the file and not in the manuscript, and that the style conforms to the rest of the article.

# Supplementary Figures and Tables

For more information on Supplementary Material and for details on the different file types accepted, please see [here](https://www.frontiersin.org/guidelines/author-guidelines#supplementary-material).

## Supplementary Figures

**
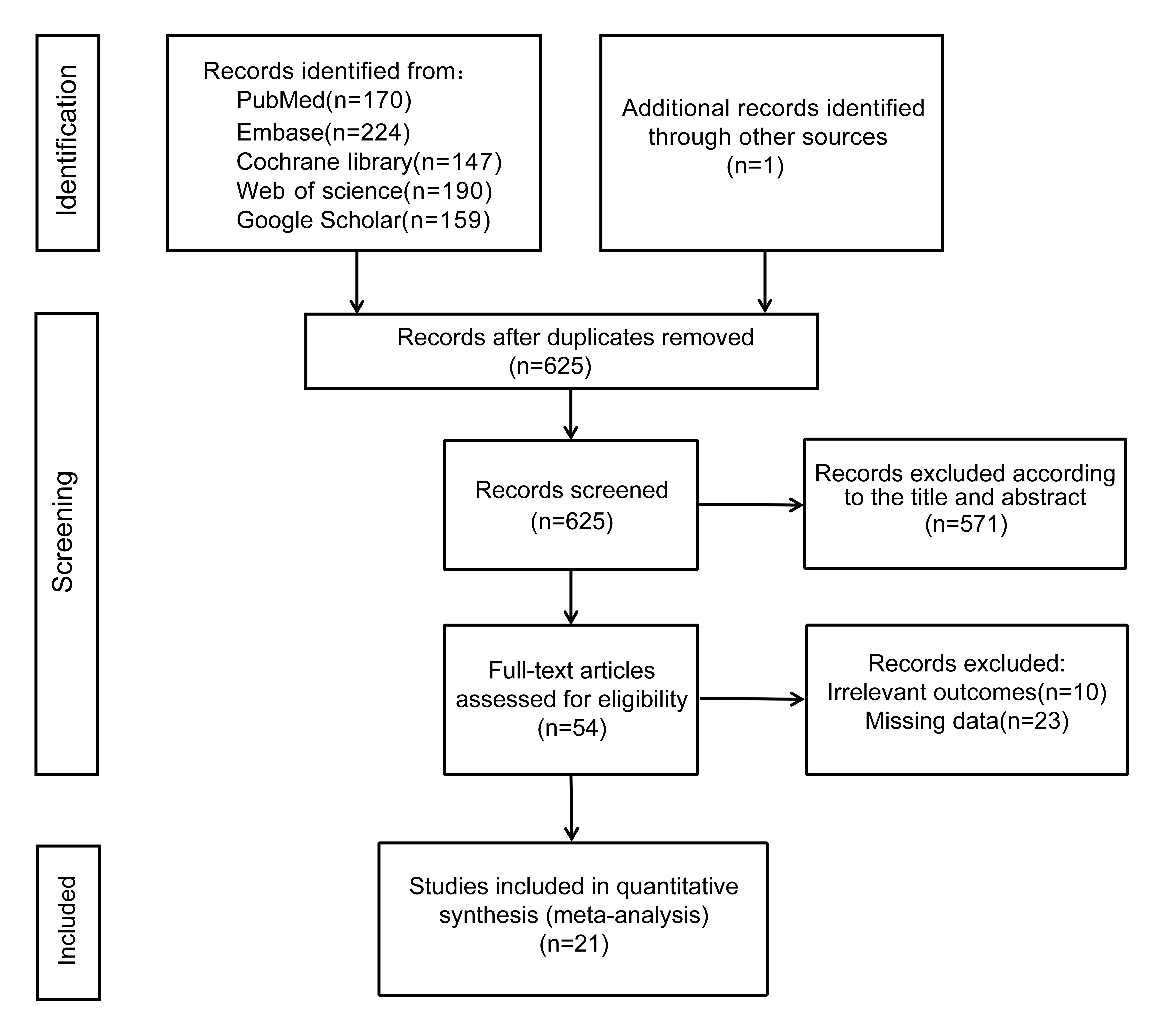
**

**Supplementary Figure 1.** Flow diagram of the literature screening process.


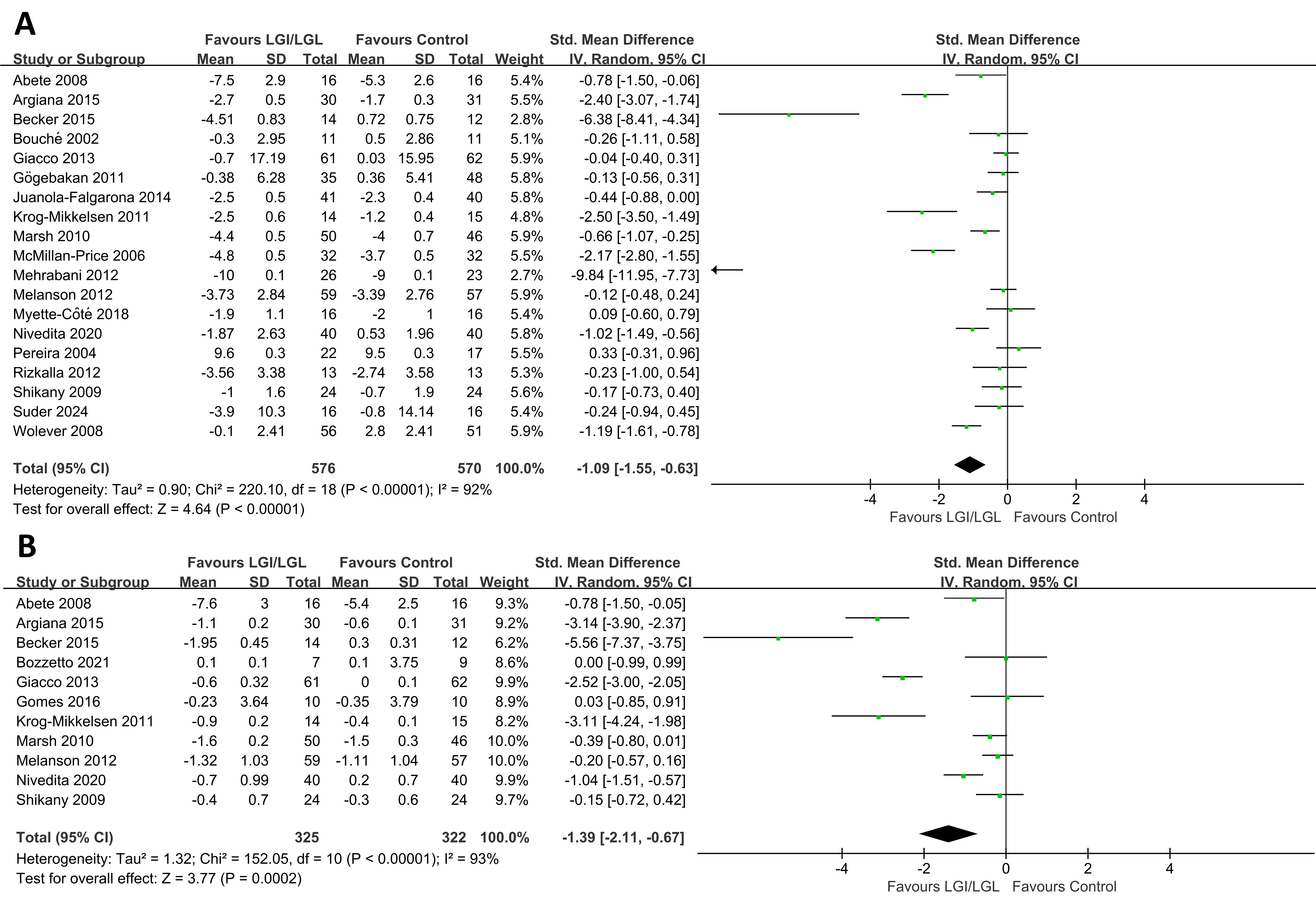


**Supplementary Figure 2**. Forest plots for the effect of LGI/LGL diets on weight loss indicators. (A) Body weight; (B) BMI**.**





**Supplementary Figure 3**. Forest plots for the effect of LGI/LGL diets on lipid metabolism indicators. (A) TC; (B) TG; (C) LDL-C; (D) HDL-C





**Supplementary Figure 4**. Forest plots for the effect of LGI/LGL diets on lipid metabolism indicators. (A) CRP; (B) TNF-α (C) IL-6; (D) APN; (E) LEP


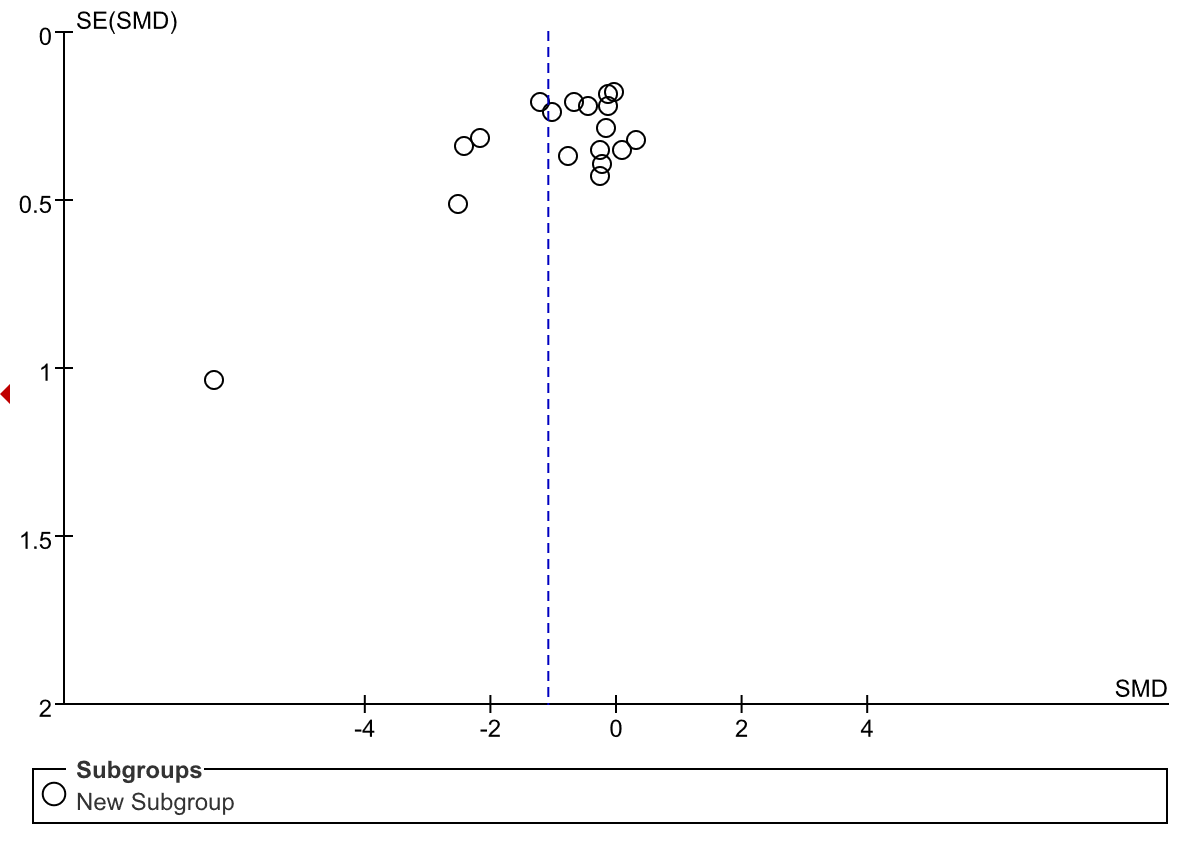


**Supplementary Figure 5**. Publication bias of Weight.


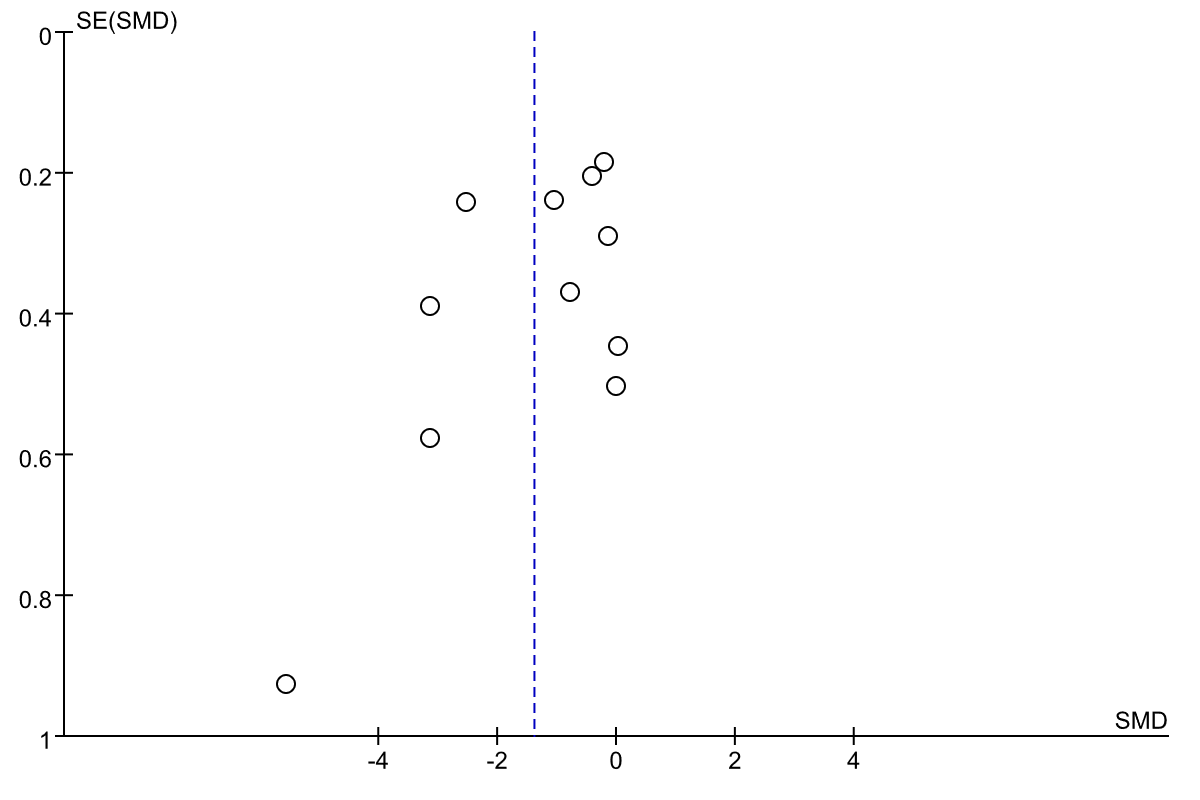


**Supplementary Figure 6**. Publication bias of BMI.


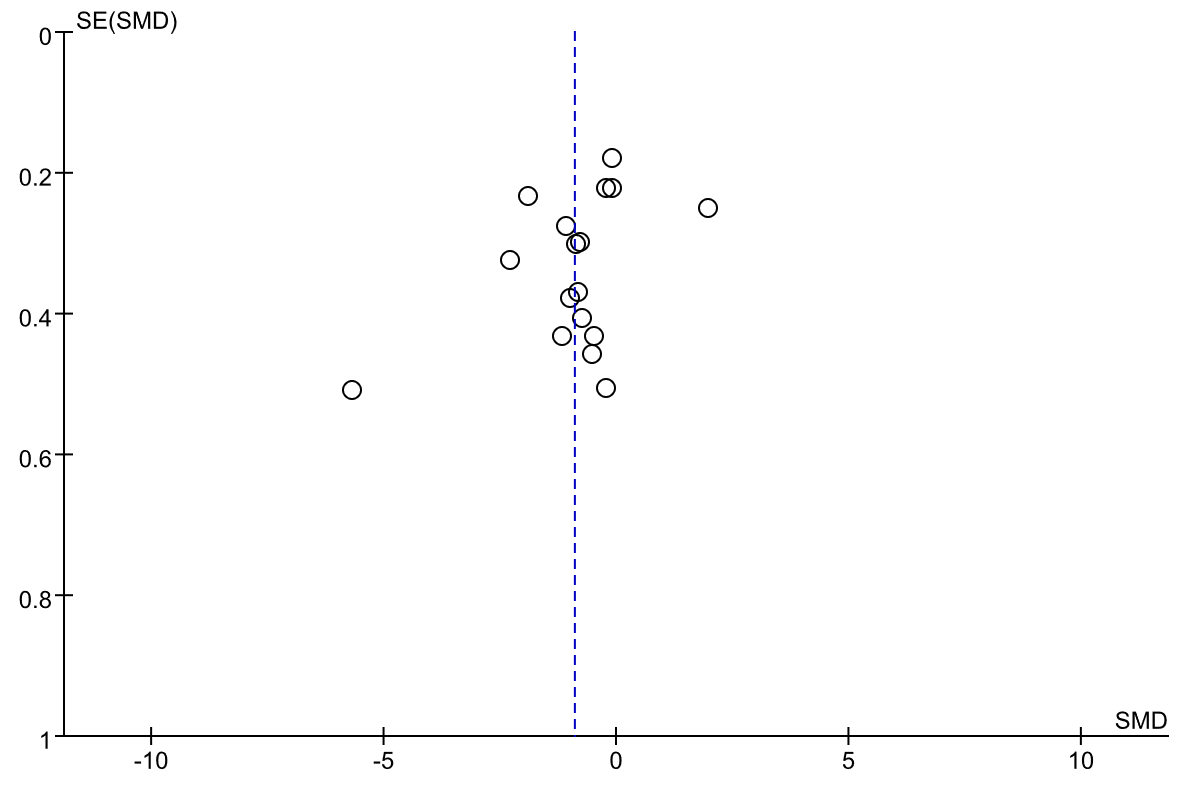


**Supplementary Figure 7**. Publication bias of TC.


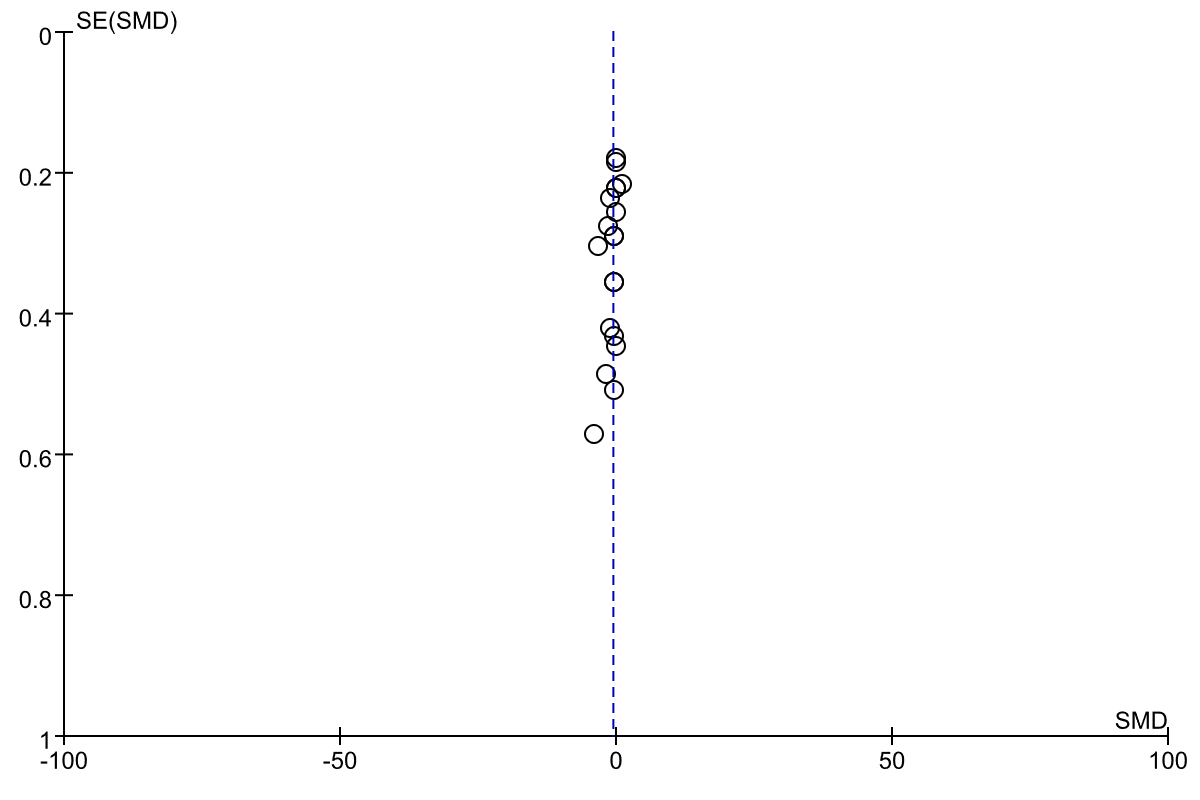


**Supplementary Figure 8**. Publication bias of TG.


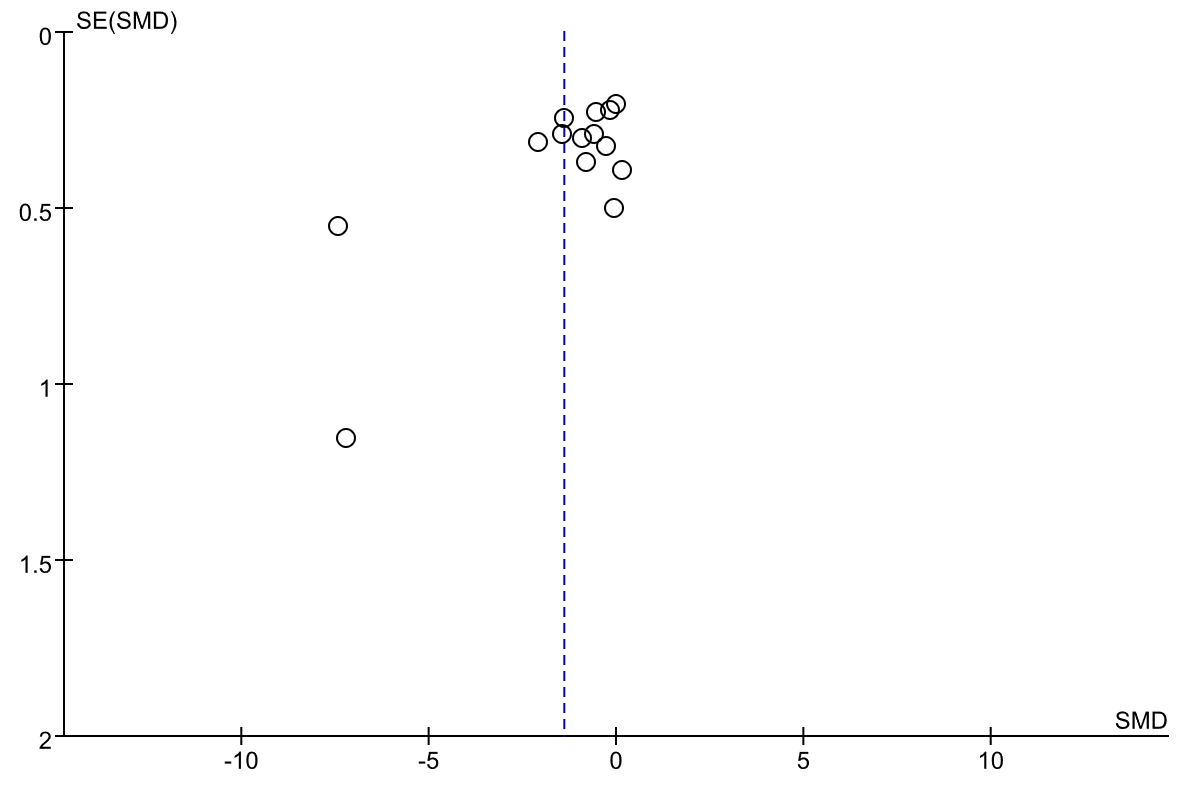


**Supplementary Figure 9**. Publication bias of LDL-C.


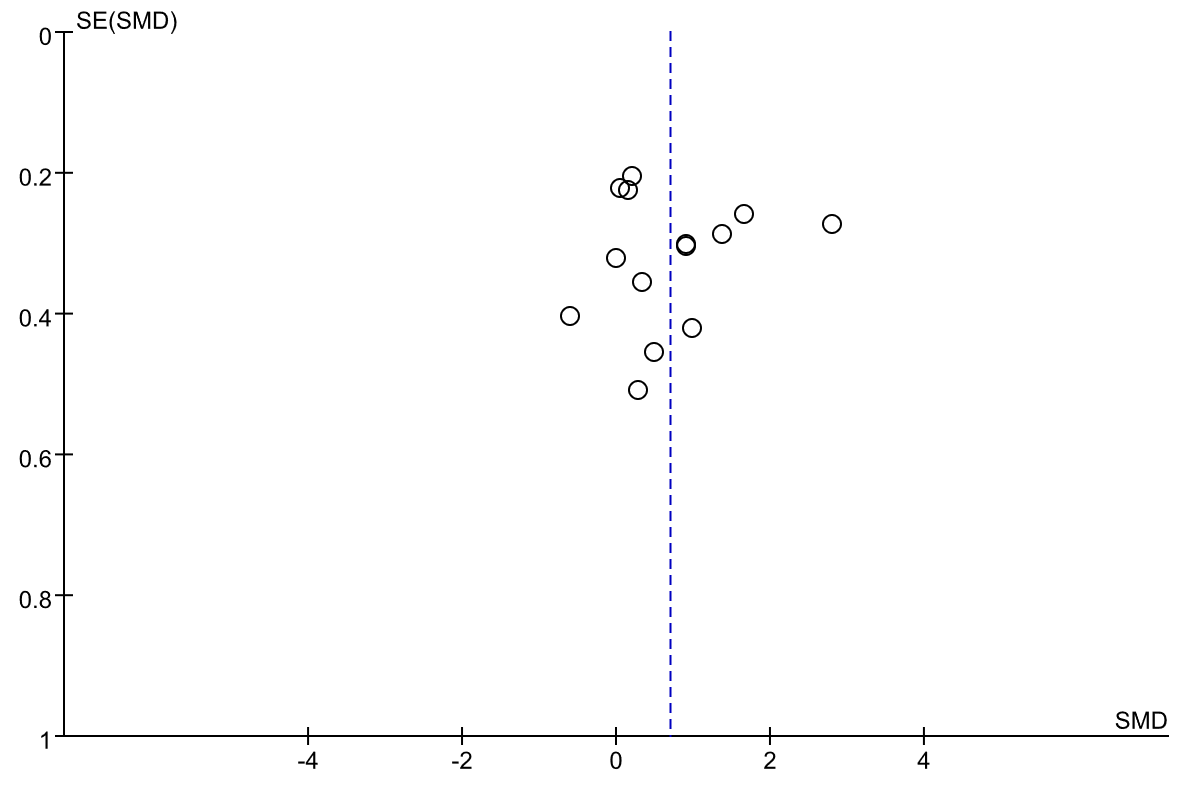


**Supplementary Figure 10**. Publication bias of HDL-C.


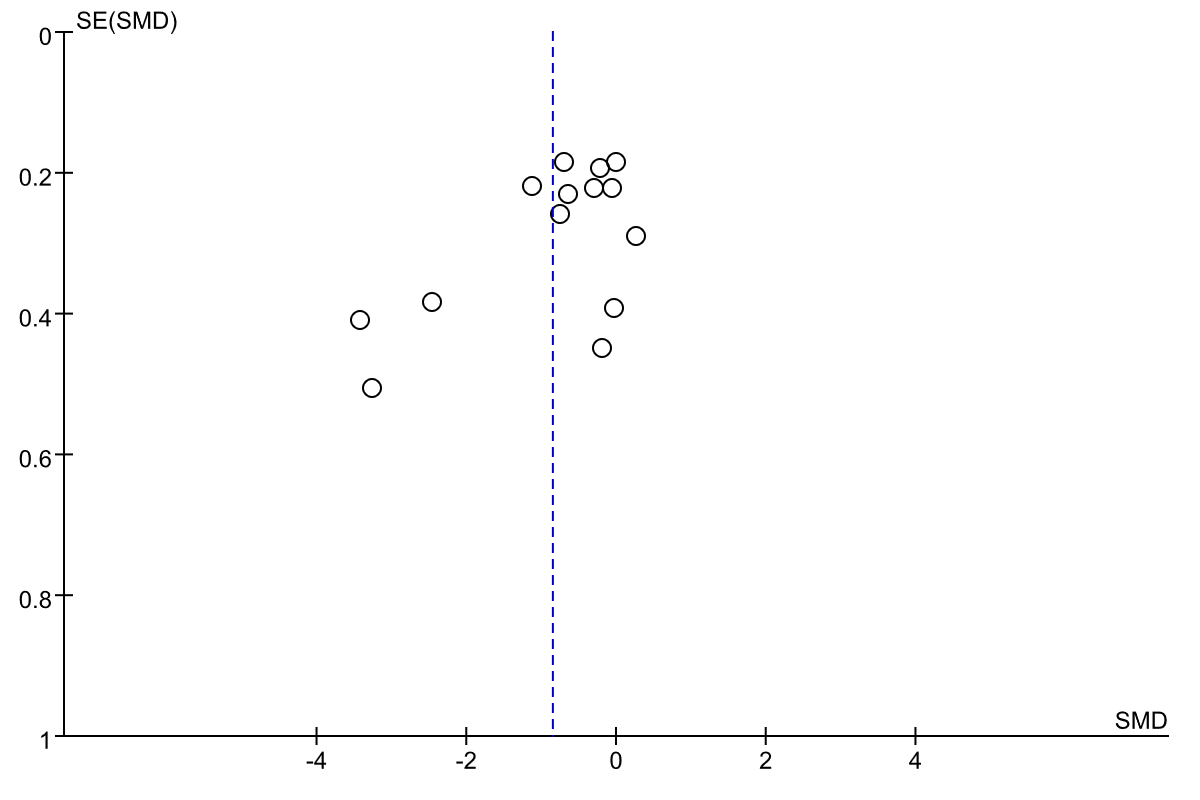


**Supplementary Figure 11**. Publication bias of CRP.


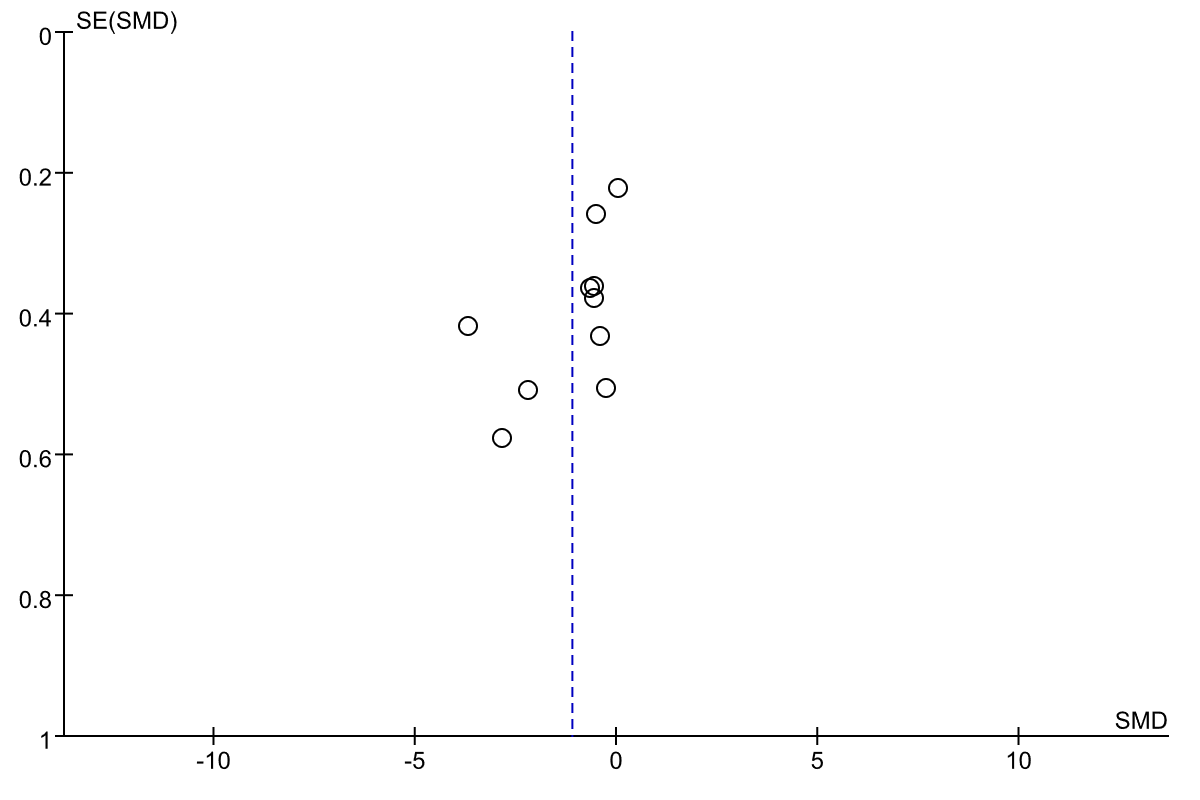


**Supplementary Figure 12**. Publication bias of Leptin.

**Supplementary Table 1.** Basic characteristics of the included studies.

| Database | Search Strategy |
| --- | --- |
| Pubmed | ("Glycemic Index"[Mesh] OR "Glycemic Load"[Mesh] OR "low glycemic index"[tiab] OR "low glycemic load"[tiab] OR "low-GI"[tiab] OR "low-GL"[tiab] OR "glycaemic index"[tiab] OR "glycaemic load"[tiab]) AND ("Inflammation"[Mesh] OR "Inflammation Mediators"[Mesh] OR "C-Reactive Protein"[Mesh] OR "Tumor Necrosis Factor-alpha"[Mesh] OR "Interleukin-6"[Mesh] OR "inflammation"[tiab] OR "inflammatory biomarker"[tiab] OR "C-reactive protein"[tiab] OR "CRP"[tiab] OR "tumor necrosis factor"[tiab] OR "TNF"[tiab] OR "interleukin-6"[tiab] OR "IL-6"[tiab] OR "leptin"[tiab] OR "adiponectin"[tiab] OR "cytokine"[tiab] OR "adipokine"[tiab]) AND ("Lipids"[Mesh] OR "Cholesterol"[Mesh] OR "Triglycerides"[Mesh] OR "Lipoproteins, LDL"[Mesh] OR "Lipoproteins, HDL"[Mesh] OR "Weight Loss"[Mesh] OR "Body Mass Index"[Mesh] OR "lipid"[tiab] OR "cholesterol"[tiab] OR "triglyceride"[tiab] OR "LDL"[tiab] OR "HDL"[tiab] OR "weight"[tiab] OR "BMI"[tiab] OR "body mass index"[tiab]) AND (randomized controlled trial[pt] OR controlled clinical trial[pt] OR randomized[tiab] OR placebo[tiab] OR drug therapy[sh] OR randomly[tiab] OR trial[tiab] OR groups[tiab]) NOT (animals[mh] NOT humans[mh]) |
| Embase | ('glycemic index'/exp OR 'glycemic load'/exp OR 'low glycemic index':ti,ab OR 'low glycemic load':ti,ab OR 'low-GI':ti,ab OR 'low-GL':ti,ab OR 'glycaemic index':ti,ab OR 'glycaemic load':ti,ab) AND ('inflammation'/exp OR 'inflammation marker'/exp OR 'c reactive protein'/exp OR 'tumor necrosis factor alpha'/exp OR 'interleukin 6'/exp OR 'leptin'/exp OR 'adiponectin'/exp OR 'inflammation':ti,ab OR 'inflammatory biomarker':ti,ab OR 'C-reactive protein':ti,ab OR 'CRP':ti,ab OR 'tumor necrosis factor':ti,ab OR 'TNF':ti,ab OR 'interleukin-6':ti,ab OR 'IL-6':ti,ab OR 'leptin':ti,ab OR 'adiponectin':ti,ab OR 'cytokine':ti,ab OR 'adipokine':ti,ab) AND ('lipid'/exp OR 'cholesterol'/exp OR 'triacylglycerol'/exp OR 'low density lipoprotein'/exp OR 'high density lipoprotein'/exp OR 'body weight loss'/exp OR 'body mass index'/exp OR 'lipid':ti,ab OR 'cholesterol':ti,ab OR 'triglyceride':ti,ab OR 'LDL':ti,ab OR 'HDL':ti,ab OR 'weight':ti,ab OR 'BMI':ti,ab OR 'body mass index':ti,ab) AND ('randomized controlled trial'/exp OR 'randomization'/exp OR 'double blind procedure'/exp OR 'single blind procedure'/exp OR 'random*':ti,ab OR 'placebo*':ti,ab OR 'trial':ti,ab) NOT ('animal'/exp NOT 'human'/exp) |
| Cochrane Library | ([mh "Glycemic Index"] OR [mh "Glycemic Load"] OR "low glycemic index":ti,ab,kw OR "low glycemic load":ti,ab,kw OR "low-GI":ti,ab,kw OR "low-GL":ti,ab,kw OR "glycaemic index":ti,ab,kw OR "glycaemic load":ti,ab,kw) AND ([mh "Inflammation"] OR [mh "Inflammation Mediators"] OR [mh "C-Reactive Protein"] OR [mh "Tumor Necrosis Factor-alpha"] OR [mh "Interleukin-6"] OR [mh "Leptin"] OR [mh "Adiponectin"] OR "inflammation":ti,ab,kw OR "inflammatory biomarker":ti,ab,kw OR "C-reactive protein":ti,ab,kw OR "CRP":ti,ab,kw OR "tumor necrosis factor":ti,ab,kw OR "TNF":ti,ab,kw OR "interleukin-6":ti,ab,kw OR "IL-6":ti,ab,kw OR "leptin":ti,ab,kw OR "adiponectin":ti,ab,kw OR "cytokine":ti,ab,kw OR "adipokine":ti,ab,kw) AND ([mh "Lipids"] OR [mh "Cholesterol"] OR [mh "Triglycerides"] OR [mh "Lipoproteins, LDL"] OR [mh "Lipoproteins, HDL"] OR [mh "Weight Loss"] OR [mh "Body Mass Index"] OR "lipid":ti,ab,kw OR "cholesterol":ti,ab,kw OR "triglyceride":ti,ab,kw OR "LDL":ti,ab,kw OR "HDL":ti,ab,kw OR "weight":ti,ab,kw OR "BMI":ti,ab,kw OR "body mass index":ti,ab,kw) |
| Web of Science | TS=("glycemic index" OR "glycemic load" OR "low glycemic index" OR "low glycemic load" OR "low-GI" OR "low-GL" OR "glycaemic index" OR "glycaemic load") AND TS=("inflammation" OR "inflammatory biomarker" OR "C-reactive protein" OR "CRP" OR "tumor necrosis factor" OR "TNF" OR "interleukin-6" OR "IL-6" OR "leptin" OR "adiponectin" OR "cytokine" OR "adipokine") AND TS=("lipid" OR "cholesterol" OR "triglyceride" OR "LDL" OR "HDL" OR "weight" OR "BMI" OR "body mass index") AND TS=("randomized controlled trial" OR "controlled clinical trial" OR "randomized" OR "placebo" OR "randomly" OR "trial") |
| Google Scholar | ("low glycemic index" OR "low glycemic load" OR "low-GI" OR "low-GL") AND (inflammation OR CRP OR TNF OR IL-6 OR leptin OR adiponectin OR cytokine OR adipokine) AND (lipid OR cholesterol OR triglyceride OR weight OR BMI) AND ("randomized controlled trial" OR "clinical trial") |

**Supplementary Table 2.** Basic characteristics of the included studies.

| First author/ year | Country/ Region | Study design | Baseline characteristics | Intervention | | | Sample size (n) | Duration | Outcomes |
| --- | --- | --- | --- | --- | --- | --- | --- | --- | --- |
|  |  |  |  | Experimental group | Control group | Other intervention group |  |  |  |
| Argiana 2015 ^(9)^ | Greece | Parallel | Males and postmenopausal females with T2DM, aged 40-65 years; BMI: 25-40 | LGI (GI: 19–49) | Medium GI (GI: 60–65) | NA | 30/31 | 12 weeks | WEIGHT, BMI, TC, TG, HDL, LDL, hs-CRP, IL-6, APN, LEP |
| Becker 2015 ^(10)^ | Brazil | Parallel | Overweight or obese infertile women, aged 18-35 years; BMI: 25-40 | LGI (GI < 55), LGL (GL < 80) | usual diets (unrestricted) | NA | 14/12 | 5 weeks | WEIGHT, TC, TG, LDL, HDL, BMI, LEP |
| Bouché 2002 ^(11)^ | France | Crossover | Healthy men, aged 46 ± 3 years | LGI diet (GI = 41.0 ± 1.0%) | HGI diet (GI = 71.3 ± 1.3%) | NA | 11 | 5 weeks | WEIGHT, TC, TG, LEP |
| Myette-Côté 2018 ^(12)^ | Britain | Crossover | Males and females with T2DM, aged 48-72 years | Low-fat LGI diet (GI = 40) | Low-carb high-fat diet (LC) | LC diet + exercise (15-min walks) | 16 | 6 weeks | WEIGHT, TG, TNF-α, IL-6 |
| Gomes 2016 ^(13)^ | Brazil | Parallel | Males and premenopausal females with T2DM, aged 42.4±5.1 years; BMI: 29.2±4.8 | LGI diet (GI: 35.8 ± 3.3) | HGI diet (GI: 74.1 ± 2.9) | NA | 10/10 | 30 days | BMI, TC, TG, HDL, APN, CRP |
| Mehrabani 2012 ^(14)^ | Iran | Parallel | Overweight or obese women, aged 20-40 years; BMI: 25-38 | Modified hypocaloric diet (prohibition of foods with GL > 20) | Conventional hypocaloric diet (CHCD) | NA | 26/23 | 12 weeks | WEIGHT, TG, TC, HDL, LDL, TNF-α, IL-6, hs-CRP, APN |
| Abete 2008 ^(15)^ | Spain | Parallel | Obese males and females, aged 36±7 years  ; BMI: 32.5±4.3 | LGI diet (GI: 40–45) | HGI diet (GI: 60–65) | NA | 16/16 | 8 weeks | WEIGHT, BMI, LEP, TC, TG, HDL, LDL |
| McMillan-Price 2006 ^(16)^ | Australia | Parallel | Overweight males and females, aged 18-40 years； BMI≥25 | LGI diet (GI: 75) | HGI diet (GI: 127) | NA | 32/32 | 12 weeks | WEIGHT, TC, TG, HDL, LDL, LEP, CRP |
| Juanola-Falgarona 2014 ^(17)^ | Spain | Parallel | Overweight or obese men and women, aged 30-60 years; MI: 27-35 | LGI diet (GI: 34) | HGI diet (GI: 62) | Low-fat diet (LF) | 41/40/40 | 6 months | WEIGHT, TC, TG, HDL, LDL, CRP, IL-6, APN, LEP |
| Krog-Mikkelsen 2011 ^(18)^ | Denmark | Parallel | Overweight females, aged 20-40 years; BMI: 25-30 | LGI diet (GI: 79) | HGI diet (GI: 103) | NA | 14/15 | 10 weeks | WEIGHT, BMI, LEP |
| Bozzetto 2021 ^(19)^ | Italy | Parallel | Males and females with T2DM | LGI diet (GI: 48) + Exercise (Ex) | Monounsaturated fatty acids (MUFA) diet (GI: 60) + Exercise (Ex) | NA | 7/9 | 8 weeks | BMI, LEP, TC, TG, HDL, LDL |
| Marsh 2010 ^(20)^ | Australia | Parallel | Healthy women, aged 18-60 years; BMI≤25 | LGI diet (GI: 40, GL: 74) | Conventional healthy diet (CHD) (GI: 74, GL: 109) | NA | 50/46 | 4 weeks | WEIGHT, BMI, TC, TG, HDL, LDL, CRP |
| Melanson 2012 ^(21)^ | America | Parallel | Overweight or obese men and women, aged 38.7±6.7 years; BMI: 31.8±2.2 | LGI diet (GI: 42.43 ± 7.35, GL: 44.75 ± 27.86) | Portion-controlled (PC) plan | Low energy density (LED) diet (GI: 40.15 ± 8.64, GL: 54.39 ± 30.14) | 59/57/41 | 12 weeks | WEIGHT, BMI, TG, HDL, CRP |
| Nivedita 2020 ^(22)^ | India | Parallel | T2DM men and women, aged 35-65 years | LGI diet | HGI diet | NA | 40/40 | 24 weeks | WEIGHT, BMI, TG, TC, HDL, LDL, hs-CRP |
| Gögebakan 2011 ^(23)^ | Europe | Parallel | Healthy men and women, aged＜65 years | LGI diet (GI: 45) | HGI diet (GI: 60) | NA | 35/48 | 26 weeks | WEIGHT, TG, TC, HDL, LDL, hs-CRP |
| Pereira 2004 ^(24)^ | America | Parallel | Overweight or obese men and women, aged 18-40 years; BMI＞27 | LGI diet | Low-fat diet | NA | 22/17 | 1 year | WEIGHT, TG, LDL, HDL, CRP |
| Rizkalla 2012 ^(25)^ | France | Crossover | Obese men and women, aged 45.0±2.4 years; BMI: 31.86±1.30 | Energy-restricted LGI diet compensated by protein (LC-P-LGI) | Conventional diet (LC-CONV) | NA | 13 | 16 weeks | WEIGHT, TC, TG, LDL, HDL, hs-CRP, TNF-α, IL-6, APN, LEP |
| Giacco 2013 ^(26)^ | Finland | Parallel | Men and women with metabolic syndrome, aged 40-65 years | Wholegrain LGI group (GI: 46) | Control HGI group (GI: 72) | NA | 61/62 | 12 weeks | WEIGHT, BMI, TC, TG, HDL, LDL, hs-CRP, TNF-α, IL-6 |
| Shikany 2009 ^(27)^ | America | Crossover | Overweight or obese men, aged 25.0±2.8 years; BMI: 29.5±4.3 | LGI diet (GI: 49.5 ± 3.3, GL: 158.3 ± 12.8) | HGI diet (GI: 75.0 ± 4.2, GL: 245.5 ± 11.4) | NA | 24 | 12 weeks | WEIGHT, BMI, TC, LDL, HDL, TG, CRP, IL-6, TNF-α |
| Suder 2024 ^(28)^ | Poland | Parallel | Abdominally obese males, aged 34.7±5.5 years; WC: 110.3±8.5cm | Exercise + High protein LGI diet (EDG) | Exercise group (EG) | Control group (CG) | 16/16/12 | 6 weeks | WEIGHT, TC, LEP |
| Wolever 2008 ^(29)^ | Canada | Parallel | T2DM men and women, aged 35-75 years; BMI: 24-40 | LGI diet (GI: 55) | HGI diet (GI: 63) | Low cholesterol diet (CHO) | 56/51/54 | 1 year | WEIGHT, TC, TG, HDL, LDL, CRP |

Note: NA, not applicable; indicates the absence of a third intervention group in two-arm studies or the non-existence of a specific subgroup for a particular outcome. For parallel designs, sample sizes are presented as experimental / control (/ other intervention). For crossover designs, the number represents the total number of participants who completed the trial. Co-interventions (e.g., exercise or specific macronutrient adjustments) were consistently applied across groups within the respective trials to minimize potential confounding effects

**Supplementary Table 3.** Risk of bias assessment.

| First author, year | Random sequence generation | Allocation concealment | Blinding of participants and personnel | Blinding of outcome assessment | Incomplete outcome data | Selective reporting | Other bias |
| --- | --- | --- | --- | --- | --- | --- | --- |
| Argiana 2015 ^(9)^ | Low | Unclear | Low | High | Low | Low | Low |
| Becker 2015 ^(10)^ | Low | Unclear | High | High | Low | Low | Low |
| Bouché 2002 ^(11)^ | Low | Unclear | Low | High | Low | Low | Low |
| Myette-Côté 2018 ^(12)^ | Low | Unclear | High | High | Low | Low | Low |
| Gomes 2016 ^(13)^ | Low | Unclear | Low | High | Low | Low | Low |
| Mehrabani 2012 ^(14)^ | Low | Unclear | Low | High | Low | Low | Low |
| Abete 2008 ^(15)^ | Low | Unclear | High | High | Low | Low | Low |
| McMillan-Price 2006 ^(16)^ | Low | Unclear | Low | High | Low | Low | Low |
| Juanola-Falgarona 2014 ^(17)^ | Low | Unclear | Low | High | Low | Low | Low |
| Krog-Mikkelsen 2011 ^(18)^ | Low | Unclear | Low | High | Low | Low | Low |
| Bozzetto 2021 ^(19)^ | Low | Unclear | Low | High | Low | Low | Low |
| Marsh 2010 ^(20)^ | Low | Unclear | High | High | High | Low | Low |
| Melanson 2012 ^(21)^ | Low | Unclear | High | High | Low | Low | Low |
| Nivedita 2020 ^(22)^ | Low | Unclear | High | High | Low | Low | Low |
| Gögebakan 2011 ^(23)^ | Low | Unclear | Low | High | Low | Low | Low |
| Pereira 2004 ^(24)^ | Low | Unclear | High | High | Low | Low | Low |
| Rizkalla 2012 ^(25)^ | Low | Unclear | Low | High | Low | Low | Low |
| Giacco 2013 ^(26)^ | Low | Unclear | High | High | Low | Low | Low |
| Shikany 2009 ^(27)^ | Low | Unclear | Low | High | Low | Low | Low |
| Suder 2024 ^(28)^ | Low | Unclear | High | High | Low | Low | Low |
| Wolever 2008 ^(29)^ | Low | Unclear | Low | Low | Low | Low | Low |

**Supplementary Table 4.** Subgroup analysis for LDL-C.

| Subgroup | Number of studies | SMD (95% CI) | *p*-value | *I^2^* (%) | Model | Between-group *p*-value |
| --- | --- | --- | --- | --- | --- | --- |
| Health status of the study population |  |  |  |  |  | 0.002 |
| Metabolic conditions | 5 | -1.85[-3.51,-0.19] | 0.03 | 98 | Random-effects |  |
| Overweight/obese | 8 | -1.22[-1.91,-0.52] | ＜0.001 | 88 | Random-effects |  |
| Healthy individuals | 2 | -0.07[-0.37,0.22] | 0.62 | 0 | Fixed-effects |  |
| Dietary patterns of the control group |  |  |  |  |  | 0.12 |
| HGI/HGL diets | 9 | -1.76[-2.74,-0.78] | ＜0.001 | 96 | Random-effects |  |
| Other dietary types / Other types of diets | 6 | -0.75[-1.58,0.09] | 0.08 | 88 | Random-effects |  |

Note: SMD, standardized mean difference; CI, confidence interval; NA, not applicable. "NA" indicates the non-existence of a specific subgroup for this outcome, or that heterogeneity and between-group p-values could not be calculated due to an insufficient number of studies (n < 2). *p* < 0.05 was considered statistically significant.

**Supplementary Table 5.** Subgroup analysis for WEIGHT.

| Subgroup | Number of studies | SMD (95% CI) | *p*-value | *I^2^* (%) | Model | Between-group *p*-value |
| --- | --- | --- | --- | --- | --- | --- |
| Health status of the study population |  |  |  |  |  | 0.19 |
| Metabolic conditions | 5 | -0.90[-1.68,-0.13] | 0.02 | 92 | Random-effects |  |
| Overweight/obese | 11 | -0.97[-1.61,-0.34] | 0.003 | 90 | Random-effects |  |
| Healthy individuals | 3 | -0.39[-0.67,-0.11] | 0.007 | 36 | Fixed-effects |  |
| Dietary patterns of the control group |  |  |  |  |  | 0.41 |
| HGI/HGL diets | 11 | -0.97[-1.46,-0.48] | ＜0.001 | 89 | Random-effects |  |
| Other dietary types / Other types of diets | 8 | -1.54[-2.52,-0.56] | 0.002 | 94 | Random-effects |  |

Note: SMD, standardized mean difference; CI, confidence interval; NA, not applicable. "NA" indicates the non-existence of a specific subgroup for this outcome, or that heterogeneity and between-group p-values could not be calculated due to an insufficient number of studies (n < 2). *p* < 0.05 was considered statistically significant.

**Supplementary Table 6.** Subgroup analysis for BMI.

| Subgroup | Number of studies | SMD (95% CI) | *p*-value | *I^2^* (%) | Model | Between-group *p*-value |
| --- | --- | --- | --- | --- | --- | --- |
| Health status of the study population |  |  |  |  |  | 0.05 |
| Metabolic conditions | 5 | -1.37[-2.49,-0.24] | 0.02 | 93 | Random-effects |  |
| Overweight/obese | 5 | -1.67[-2.85,-0.49] | 0.006 | 93 | Random-effects |  |
| Healthy individuals | 1 | -0.39[-0.80,0.01] | 0.06 | NA | NA |  |
| Dietary patterns of the control group |  |  |  |  |  | 0.53 |
| HGI/HGL diets | 7 | -1.51[-2.43,-0.59] | 0.001 | 93 | Random-effects |  |
| Other dietary types / Other types of diets | 4 | -1.06[-2.10,-0.02] | 0.05 | 91 | Random-effects |  |

Note: SMD, standardized mean difference; CI, confidence interval; NA, not applicable. "NA" indicates the non-existence of a specific subgroup for this outcome, or that heterogeneity and between-group p-values could not be calculated due to an insufficient number of studies (n < 2). *p* < 0.05 was considered statistically significant.

**Supplementary Table 7.** Subgroup analysis for TG.

| Subgroup | Number of studies | SMD (95% CI) | *p*-value | *I^2^* (%) | Model | Between-group *p*-value |
| --- | --- | --- | --- | --- | --- | --- |
| Health status of the study population |  |  |  |  |  | 0.12 |
| Metabolic conditions | 7 | -0.70[-1.60,0.20] | 0.13 | 94 | Random-effects |  |
| Overweight/obese | 9 | -0.89[-1.47,-0.31] | 0.003 | 88 | Random-effects |  |
| Healthy individuals | 3 | 0.19[-0.68,1.07] | 0.66 | 88 | Random-effects |  |
| Dietary patterns of the control group |  |  |  |  |  | 0.72 |
| HGI/HGL diets | 8 | -0.77[-1.59,0.04] | 0.06 | 92 | Random-effects |  |
| Other dietary types / Other types of diets | 11 | -1.06[-2.10,-0.02] | 0.05 | 92 | Random-effects |  |

Note: SMD, standardized mean difference; CI, confidence interval; NA, not applicable. "NA" indicates the non-existence of a specific subgroup for this outcome, or that heterogeneity and between-group p-values could not be calculated due to an insufficient number of studies (n < 2). *p* < 0.05 was considered statistically significant.

**Supplementary Table 8.** Subgroup analysis for TC.

| Subgroup | Number of studies | SMD (95% CI) | *p*-value | *I^2^* (%) | Model | Between-group *p*-value |
| --- | --- | --- | --- | --- | --- | --- |
| Health status of the study population |  |  |  |  |  | 0.06 |
| Metabolic conditions | 6 | -0.66[-1.34,0.02] | 0.06 | 90 | Random-effects |  |
| Overweight/obese | 8 | -1.63[-2.55,-0.71] | ＜0.001 | 92 | Random-effects |  |
| Healthy individuals | 3 | 0.45[-1.16,2.07] | 0.58 | 96 | Random-effects |  |
| Dietary patterns of the control group |  |  |  |  |  | 0.16 |
| HGI/HGL diets | 11 | -1.24[-1.94,-0.55] | ＜0.001 | 94 | Random-effects |  |
| Other dietary types / Other types of diets | 6 | -0.27[-1.44,0.91] | 0.65 | 94 | Random-effects |  |

Note: SMD, standardized mean difference; CI, confidence interval; NA, not applicable. "NA" indicates the non-existence of a specific subgroup for this outcome, or that heterogeneity and between-group p-values could not be calculated due to an insufficient number of studies (n < 2). *p* < 0.05 was considered statistically significant.

**Supplementary Table 9.** Subgroup analysis for HDL-C.

| Subgroup | Number of studies | SMD (95% CI) | *p*-value | *I^2^* (%) | Model | Between-group *p*-value |
| --- | --- | --- | --- | --- | --- | --- |
| Health status of the study population |  |  |  |  |  | 0.12 |
| Metabolic conditions | 6 | 1.04[-0.05,2.13] | 0.06 | 93 | Random-effects |  |
| Overweight/obese | 8 | 0.56[0.07,1.06] | 0.02 | 80 | Random-effects |  |
| Healthy individuals | 2 | 0.13[-0.17,0.42] | 0.40 | 0 | Fixed-effects |  |
| Dietary patterns of the control group |  |  |  |  |  | 0.17 |
| HGI/HGL diets | 10 | 0.80[0.23,1.37] | 0.006 | 92 | Random-effects |  |
| Other dietary types / Other types of diets | 6 | 0.30[-0.14,0.74] | 0.18 | 61 | Random-effects |  |

Note: SMD, standardized mean difference; CI, confidence interval; NA, not applicable. "NA" indicates the non-existence of a specific subgroup for this outcome, or that heterogeneity and between-group p-values could not be calculated due to an insufficient number of studies (n < 2). *p* < 0.05 was considered statistically significant.

**Supplementary Table 10.** Subgroup analysis for CRP.

| Subgroup | Number of studies | SMD (95% CI) | *p*-value | *I^2^* (%) | Model | Between-group *p*-value |
| --- | --- | --- | --- | --- | --- | --- |
| Health status of the study population |  |  |  |  |  | 0.89 |
| Metabolic conditions | 5 | -0.99[-1.80,-0.18] | 0.02 | 92 | Random-effects |  |
| Overweight/obese | 7 | -0.83[-1.58,-0.08] | 0.03 | 92 | Random-effects |  |
| Healthy individuals | 2 | -0.71[-1.52,0.09] | 0.08 | 85 | Random-effects |  |
| Dietary patterns of the control group |  |  |  |  |  | 0.17 |
| HGI/HGL diets | 9 | -0.62[-1.10,-0.15] | 0.01 | 92 | Random-effects |  |
| Other dietary types / Other types of diets | 5 | -1.33[-2.39,-0.27] | 0.01 | 61 | Random-effects |  |
| CRP type |  |  |  |  |  | 0.24 |
| CRP | 8 | -1.22[-2.03,-0.40] | 0.003 | 88 | Random-effects |  |
| hs-CRP | 6 | -0.86[-1.30,-0.41] | 0.03 | 94 | Random-effects |  |

Note: SMD, standardized mean difference; CI, confidence interval; NA, not applicable. "NA" indicates the non-existence of a specific subgroup for this outcome, or that heterogeneity and between-group p-values could not be calculated due to an insufficient number of studies (n < 2). *p* < 0.05 was considered statistically significant.

**Supplementary Table 11.** Subgroup analysis for TNF-α.

| Subgroup | Number of studies | SMD (95% CI) | *p*-value | *I^2^* (%) | Model | Between-group *p*-value |
| --- | --- | --- | --- | --- | --- | --- |
| Health status of the study population |  |  |  |  |  | 0.61 |
| Metabolic conditions | 2 | -0.46[-0.78,-0.14] | 0.005 | 29 | Fixed-effects |  |
| Overweight/obese | 3 | -0.34[-0.69,0.02] | 0.06 | 0 | Fixed-effects |  |
| Healthy individuals | 0 | NA | NA | NA | NA |  |
| Dietary patterns of the control group |  |  |  |  |  | 0.37 |
| HGI/HGL diets | 2 | -0.49[-0.80,-0.19] | 0.002 | 0 | Fixed-effects |  |
| Other dietary types / Other types of diets | 3 | -0.27[-0.65,0.11] | 0.17 | 0 | Fixed-effects |  |

Note: SMD, standardized mean difference; CI, confidence interval; NA, not applicable. "NA" indicates the non-existence of a specific subgroup for this outcome, or that heterogeneity and between-group p-values could not be calculated due to an insufficient number of studies (n < 2). *p* < 0.05 was considered statistically significant.

**Supplementary Table 12.** Subgroup analysis for IL-6.

| Subgroup | Number of studies | SMD (95% CI) | *p*-value | *I^2^* (%) | Model | Between-group *p*-value |
| --- | --- | --- | --- | --- | --- | --- |
| Health status of the study population |  |  |  |  |  | 0.68 |
| Metabolic conditions | 3 | -0.68[-1.84,0.48] | 0.25 | 93 | Random-effects |  |
| Overweight/obese | 4 | -0.36[-0.64,-0.09] | 0.01 | 51 | Fixed-effects |  |
| Healthy individuals | 0 | NA | NA | NA | NA |  |
| Dietary patterns of the control group |  |  |  |  |  | 0.87 |
| HGI/HGL diets | 4 | -0.52[-1.27,0.22] | 0.17 | 90 | Random-effects |  |
| Other dietary types / Other types of diets | 3 | -0.59[-0.98,-0.19] | 0.003 | 43 | Fixed-effects |  |

Note: SMD, standardized mean difference; CI, confidence interval; NA, not applicable. "NA" indicates the non-existence of a specific subgroup for this outcome, or that heterogeneity and between-group p-values could not be calculated due to an insufficient number of studies (n < 2). *p* < 0.05 was considered statistically significant.

**Supplementary Table 13.** Subgroup analysis for APN.

| Subgroup | Number of studies | SMD (95% CI) | *p*-value | *I^2^* (%) | Model | Between-group *p*-value |
| --- | --- | --- | --- | --- | --- | --- |
| Health status of the study population |  |  |  |  |  | 0.97 |
| Metabolic conditions | 2 | 0.27[-0.94,1.48] | 0.67 | 81 | Random-effects |  |
| Overweight/obese | 3 | 0.29[-0.10,0.67] | 0.14 | 0 | Fixed-effects |  |
| Healthy individuals | 0 | NA | NA | NA | NA |  |
| Dietary patterns of the control group |  |  |  |  |  | 0.22 |
| HGI/HGL diets | 3 | 0.01[-0.36,0.39] | 0.94 | 62 | Fixed-effects |  |
| Other dietary types / Other types of diets | 2 | 0.38[-0.08,0.85] | 0.10 | 25 | Fixed-effects |  |

Note: SMD, standardized mean difference; CI, confidence interval; NA, not applicable. "NA" indicates the non-existence of a specific subgroup for this outcome, or that heterogeneity and between-group p-values could not be calculated due to an insufficient number of studies (n < 2). *p* < 0.05 was considered statistically significant.

**Supplementary Table 14.** Subgroup analysis for LEP.

| Subgroup | Number of studies | SMD (95% CI) | *p*-value | *I^2^* (%) | Model | Between-group *p*-value |
| --- | --- | --- | --- | --- | --- | --- |
| Health status of the study population |  |  |  |  |  | 0.21 |
| Metabolic conditions | 2 | -0.45[-0.91,0.00] | 0.05 | 0 | Fixed-effects |  |
| Overweight/obese | 7 | -1.43[-2.46,-0.40] | 0.006 | 93 | Random-effects |  |
| Healthy individuals | 1 | -0.41[-1.26,0.43] | 0.34 | NA | NA |  |
| Dietary patterns of the control group |  |  |  |  |  | 0.26 |
| HGI/HGL diets | 7 | -0.84[-1.67,-0.01] | 0.05 | 91 | Random-effects |  |
| Other dietary types / Other types of diets | 3 | -1.79[-3.20,-0.37] | 0.01 | 85 | Random-effects |  |

Note: SMD, standardized mean difference; CI, confidence interval; NA, not applicable. "NA" indicates the non-existence of a specific subgroup for this outcome, or that heterogeneity and between-group p-values could not be calculated due to an insufficient number of studies (n < 2). *p* < 0.05 was considered statistically significant.
